# A New Class of Glucosyl Thioureas: Synthesis and Larvicidal Activities

**DOI:** 10.3390/molecules21070925

**Published:** 2016-07-16

**Authors:** Ping-An Wang, Jun-Tao Feng, Xing-Zi Wang, Mu-Qiong Li

**Affiliations:** 1 Department of Medicinal Chemistry, School of Pharmacy, The Fourth Military Medical University, Changle West Road 169, Xi’an 710032, China; 13299079818@163.com (X.-Z.W.); limuqiong1981@163.com (M.-Q.L.); 2 Research & Department Center of Biorational Pesticides, Northwest Agriculture & Forestry University, No. 3 Taicheng Road, Yangling 712100, China; jtfeng@126.com

**Keywords:** glucosyl thiourea, chitin synthase inhibitor, anti-chitin structure, larvicidal activity, growth inhibitory rate

## Abstract

A novel series of glucosyl thioureas were synthesized in good overall yields (up to 37% over four steps) from d-glucose and primary amines, and their larvicidal activities toward *Mythimna separata* Walker were also investigated. This new class of glucosyl thioureas demonstrated low to moderate growth inhibition activity of *Mythiman separata* Walker, with a growth inhibitory rate of up to 47.5% at a concentration of 100.0 mg/L in acetone.

## 1. Introduction

Numerous traditional pesticides are banned in many countries because of their high toxicity [1,2,3,4,5] and poor biodegradation [6,7,8,9,10,11]. Fortunately, green pesticides [12,13,14,15] for mimic natural pesticides [16,17,18,19,20] are efficient strategies in pesticide research and development, and pesticides from natural resources are very attractive to academic and industrial researchers due to their unique pesticidal activities, good biodegradation and environmental friendliness. Chitin is the most abundant natural polyaminosaccharide and serves as a structural component to form the skin of many arthropod pests. Chitin synthesis is controlled by chitin synthase, and if this synthesis is interrupted, arthropod pests cannot survive without skin. Due to lack of an equivalent chitin synthesis in plants and vertebrates, the chitin synthesis process is considered as an ideal and safe target site for pesticide development. Over past four decades, a lot of compounds have been successfully launched worldwide as chitin synthesis inhibitors (CSIs) [21,22,23,24] to combat arthropod pests in agriculture and forestry. Among them, benzoylureas such as diflubenzuron [25,26,27] and novaluron [28,29] are the most popular chemicals among commercially available pesticides, and they are also regarded as green pesticides. Unfortunately, benzoylurea residues have been detected in recent years in soils, rivers, crops and fruits, and it can be considered as low biodegradability products with potential hazards to human health, and flufenoxuron, one of the benzoylureas, was banned in the European Union in 2011 due to its high risk to aquatic organisms and high potential for bioaccumulation in the food chain. Although the mode of action of chitin synthesis inhibitors is somewhat elusive [23], the research and development of novel CSIs with high efficiency and good biodegradation are still attractive for pesticide development.

**Figure 1:**
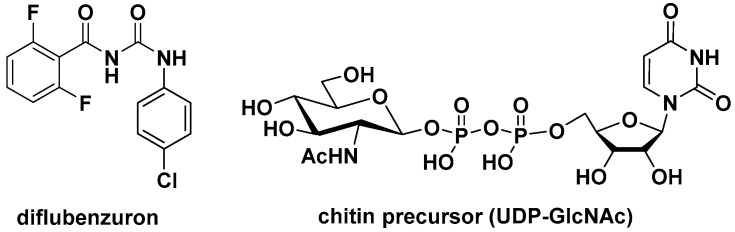
The structures of diflubenzuron and chitin precursor.

**Figure 2:**
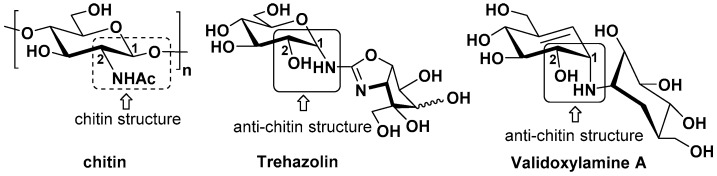
The “anti-chitin structure” of some natural CSIs.

**Figure 3:**
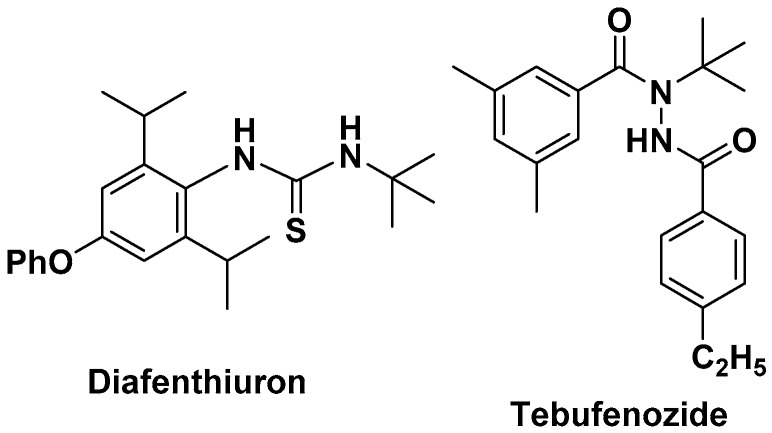
The structures of diafenthiuron and tebufenozide.

Carbohydrates and amino acids are common biomolecules in living organisms and play key roles in many important biological activities. Both of them are easily biodegradable. Some pesticides are derived from or contain carbohydrates and peptides. The combination of these two biomolecules for the exploration of pesticides is a new trend in recent years [30,31,32,33,34]. Uridine diphosphate *N*-acetyl-β-glucosamine (UDP-GlcNAc), the precursor for chitin synthesis, can be divided into two parts, one part is *N*-acetyl-β-glucosamine (GlcNAc) portion, and the other part is uridine diphosphate (UDP). These two parts are connected by β-(1-4)-glycosidic bonds (Figure 1). Firstly, due to the lack of structural information about chitin synthase, the details of the chitin synthesis process remain elusive, but researchers have found that β-(1-4)-glycosidic bond formation between aminosaccharides with inversion of the configuration at the anomeric center occurs in the transfer of GlcNAc residues to chitin [23]. Natural CSIs such as trehazolin [35,36,37,38], salbostatin [39,40,41], and validoxylamine [42,43,44], possess a similar structure to UDP-GlcNAc, so we can hypothesize that chitin synthase perhaps mistakes these natural CSIs for chitin synthetic precursors to complete chitin synthesis resulting in interruption of chitin formation. Secondly, in the GlcNAc part of the chitin precursor, the hemiacetal hydroxyl group and amino group are located on the 1- and 2- positions of the glucose ring, whereas these two groups are arranged at 2- and 1- positions in the sugar ring of the abovementioned natural CSIs. Such opposite location of two groups in these above chemicals is called the “anti-chitin structure” (Figure 2). We believe that chitin synthase probably follows a “like uses like” principle, and misuses these “anti-chitin structure” chemicals as chitin precursors for chitin synthesis and this leads to the discontinuation of chitin synthesis. The thiourea group is used as a functional group for some pesticides to achieve highly efficient larvicidal activity, for instance, diafenthiuron [45,46,47,48] containing one thiourea moiety shows a strong larvicidal ability toward mites (Figure 3). With this in mind, inclusion of a thiourea group is a feature of the designed molecules presented in this paper. In order to validate the above hypothesis, herein we have synthesized a novel series of glucosyl thioureas with “anti-chitin structure” from d-glucose and amino acids or amines, and their preliminary pesticidal and growth inhibitory activities to *Mythiman separata* Walker have also been investigated. 

## 2. Results and Discussion

### 2.1. Chemical Synthesis of d-Glucose-Based Thioureas

**Scheme 1:**
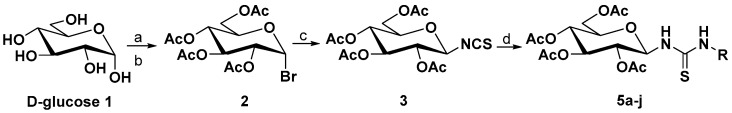
General synthetic route to glucose-based thioureas **5**. *Reagents and Conditions*: (**a**) Ac_2_O, one drop of conc. HClO_4_, −5~0 °C; (**b**) Br_2_, dropwise, −5~0 °C, 57% (2 steps); (**c**) 1.1 eq. Pb(SCN)_2_, toluene, 80 °C, 82%; (**d**) RNH_2_ (**4a**–**j**), CH_2_Cl_2_, r.t., 81%~96%.

The chemical synthesis of novel “anti-chitin structural” thioureas (Scheme 1) begins from d-glucose (**1**). The acetylation of d-glucose following substitution of one of the hemiacetal position acetate groups of glucose with bromine affords 1-bromo-2,3,4,6-tetraacetyl-d-glucose (**2**) in moderate (57%) yield. Intermediate **2** reacts with an excess of Pb(SCN)_2_ in hot toluene to furnish isothiocyanate **3** in 82% yield. The intermediate **3** should be quickly due to its instability when exposed to air and light.

With peracetoxy-d-glucosyl isothiocyanate **3** in hand, the ethyl ester of l-phenylalanine (**4a**) was chosen as a chiral amine for coupling with **3** in anhydrous CH_2_Cl_2_ at room temperature (r.t.) to give “anti-chitin structural” thiourea **5a** in high yield (91%). Following the same method, the other thioureas **5b**–**j** were also prepared in good yields. For instance, the ethyl esters of d-phenylalanine (**4b**), l-phenylalaninol (**4c**), d-phenylalaninol (**4d**), (*S*)-α-methylbenzylamine (**4e**), (*R*)-α-methyl-benzylamine (**4f**) and benzylamine (**4g**) were introduced into the molecules to afford thioureas **5b**–**g** for investigation of the effect on pesticidal activities with the change of the stereogenic center of the amine groups. Some previous studies suggest that the existence of hydrogen-binding interactions between chitin synthase and pesticidal molecules can enhance the pest-controling bioactivity [23]. Therefore, l-phenylalaninol (**4c**) and d-phenylalaninol (**4d**) were used for the construction of these “anti-chitin structural” glycosyl thioureas **5c** and **5d** to produce many more hydrogen-binding interactions. Tebufenozide [49,50,51,52,53] is a best selling pesticide in recent decades which contains one benzoylhydrazine group (Figure 3), so benzoylhydrazine (**4h**) was anchored in thiourea **5h**. Thioureas **5i** and **5j** contain quinine and taxol C-13 side moieties, respectively. These two groups are derived from natural products, and hopefully would afford good pesticidal activities. All these prepared glycosyl thioureas **5a**–**j** were fully characterized by their NMR and MS spectra (in Supplementary Matrials). This synthetic route enjoys many advantages such as commercially available materials, easily scalable procedures and highly reproducible yields.

### 2.2. Pesticidal and Growth Inhibition Actvities of d-Glucose-Based Thioureas

**Figure 4:**
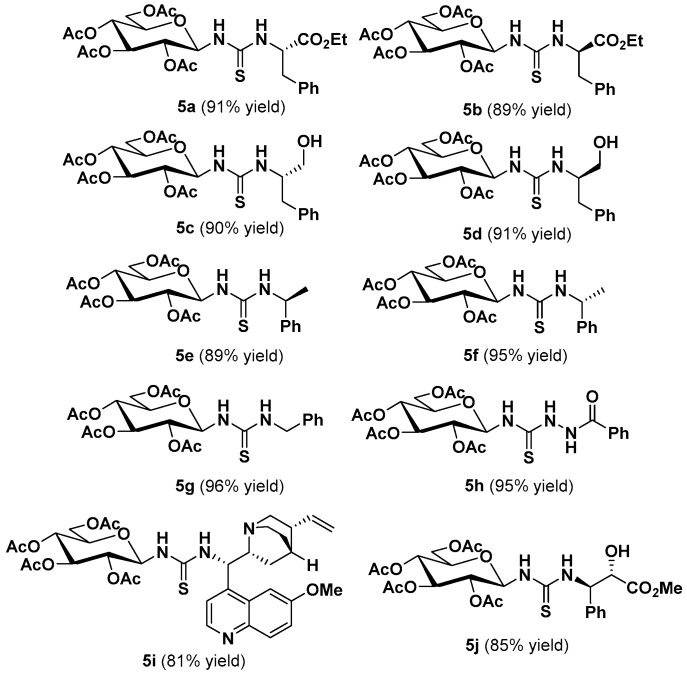
The structures of d-glucosyl thioureas **5a**–**j**.

Firstly, the larvicidal activities of these “anti-chitin structural” glycosyl thioureas **5a**–**j** (Figure 4) against oriental armyworm (*Mythimna separata* Walker) were investigated [54,55,56,57]. All of the compounds were tested at various concentrations from 6.25 mg/L to 100 mg/L, and the results are summarized in Table 1. From the results we can see that at the concentration of 6.25 mg/L, none of the tested thioureas showed any larvicidal activity. As the concentrations increased, the larvicidal activities also increased, but unfortunately, no highly larvicidal activities were found for any of the tested thioureas, even at the highest concentration of 100 mg/L. Thioureas **5e**, **5f** (containing an α-methylbenzylamino group), **5g** (with a benzylamino group), **5i** (possessing a quininylamino group) and **5j** (from the C-13 side chain of taxol) demonstrated no larvicidal activities at the concentration of 100 mg/L, and the compound **5h** (with a benzoylhydrazine group) only showed slightly larvicidal activity under the same conditions. To our delight, thioureas **5a**–**5d** (derived from phenylalanine) exhibited low larvicidal activities to *Mythiman separata* Walker at the concentration of 100.0 mg/L.

During our investigation of the larvicidal activities of these thioureas, we found that the growth rate of the pests treated by compounds **5a**, **5b** and **5c** are significantly slower than those of the control group (CG). This phenomenon indicated that these four thioureas possess growth inhibition activities towards *Mythiman separata* Walker. To this point, the growth inhibition of these four “anti-chitin structural” glycosyl thioureas was tested, and the results are listed in Table 2, where it is found that the growth inhibitory rates of **5a** and **5b** are 39.34% and 34.75% at the concentration of 100.0 mg/L, respectively. Thiourea **5c** derived from d-glucosyl isothiocyanate and l-phenylalaninol showed the best growth inhibitory ability (up to 47.38%) on *Mythiman separata* Walker. This is probably due to the existence of hydrogen-binding interactions between chitin synthase and the two NH and one hydroxyl groups of compound **5c**.

## 3. Materials and Methods 

### 3.1. General Information 

Melting points are uncorrected and expressed in °C. ^1^H-NMR (400 MHz) and ^13^C-NMR (100 MHz) spectra were measured in CDCl_3_ solution on an AV-400 spectrometer (Bruker, USA) using TMS as an internal reference. Coupling constant (*J*) values are given in Hz. Multiplicities are designated by the following abbreviations: s, singlet; d, doublet; t, triplet; q, quartet; br, broad; m, multiplet. High-resolution mass spectra were performed on a Bruker microTOF-Q II Mass Spectrometer with ES ionization (ESI). All commercially available reagents were used as received. Products were purified by flash column chromatography on silica gel (200–300 mesh) purchased from Qingdao Hai Yang Chemical Co., Ltd. (Qingdao, China). Reagents were all analytically pure. All solvents and liquid reagents were dried by standard methods in advance and distilled before use. All reactions involving air or moisture sensitive species were performed in oven-dried glassware under inert atmosphere. 1-Bromo-2,3,4,6-tetraacetyl-d-glucose (**2**) [58], isothiocyanate **3** [59,60], (9*S*)-aminoquinine (**4i**) [61,62] and the C-13 side chain of taxol **4j** [63,64] were prepared according to the corresponding literature methods. 

### 3.2. Typical Procedure for the Preparation of ***5a**–**j***: Preparation of (2S)-((2,3,4,6-tetra-O-Acetyl-β-d-gluco-pyranosyl)carbamothioylamino)-3-phenyl-1-propanol (***5c***)

Under an inert atmosphere l-phenylalaninol (**4c**, 1.52 g, 5.05 mmol) in anhydrous CH_2_Cl_2_ (10 mL) added dropwise by syringe over 5 min at room temperature to a solution of isothiocyanate **3** (3.90 g, 5.0 mmol) in anhydrous CH_2_Cl_2_ (15.0 mL). The resulting mixture was stirred for 3 h and reaction progress was monitored by thin-layer chromatography (eluent: *n*-hexane/EtOAc = 5:1, *v*/*v*). When the reaction was finished, the solvent was removed under reduced pressure to give a yellow foam which was directly purified by a flash column chromatography (eluent: *n*-hexane/EtOAc = 5:1, *v*/*v*) to afford 2.44 g (90%) of the pure isothiocyanate **5c** as light yellow crystals, m.p. 79.2–81.4 °C; *R*_f_ (*n*-hexane/EtOAc = 5:1): 0.40; [α${]}_{D}^{25}$ − 29.6 (*c* 0.5, CHCl_3_); ^1^H-NMR (CDCl_3_): δ 7.34–7.19 (m, 5H), 7.01–6.99 (m, 1H), 5.62 (br, 1H), 5.37–5.32 (m, 1H), 5.15–5.02 (m, 2H), 4.35–4.28 (m, 1H), 4.16–4.08(m, 1H), 3.87–3.85 (m, 1H), 3.74–3.71 (m, 1H), 3.60–3.57 (m, 1H), 2.95–2.68 (m, 3H), 2.17 (m, 1H), 2.05 (m, 13 H, 4 CH_3_ + 1H); ^13^C-NMR (CDCl_3_): δ 183.44, 171.45, 170.91, 170.80, 169.93, 169.72, 137.27, 129.24, 128.71, 126.81, 80.20, 73.36, 72.84, 70.85, 68.29, 61.70, 38.80, 36.59, 28.16, 20.78, 20.62, 20.60; MS (ESI): *m*/*z* (%): 563.1 [M + Na]^+^ (100); HRMS (ESI, *m*/*z*): Calcd for C_24_H_33_N_2_O_10_S [M + H]^+^: 541.1856, Found: 541.1854.

The following isothiocyanates were similarly prepared following this general procedure:

*Ethyl (2,3,4,6-tetra-O-acetyl-β-d-glucopyranosyl)carbamothioyl-l-phenyl alaninate* (**5a**) [65]. Syrup (2.65 g, 91%); Lit. [65] light yellow solid, m.p. 86.7–88.5 °C; *R*_f_ (*n*-hexane/EtOAc = 5:1): 0.45; [α${]}_{D}^{25}$ + 35.6 (*c* 0.5, CHCl_3_); ^1^H-NMR (CDCl_3_): δ 7.28–7.26 (m, 2H), 7.12–7.10 (m, 2H), 6.95–6.90 (m, 2H), 5.60 (br, 1H), 5.33 (t, *J* = 8.8 Hz, 2H), 5.07 (t, *J* = 8.8 Hz, 1H), 4.97 (t, *J* = 9.2 Hz, 1H), 4.36–4.33 (m, 1H), 4.15 (q, *J* = 6.8 Hz, 2H), 4.06 (d, *J* = 12.4 Hz, 1H), 3.81–3.79 (m, 1H), 3.30–3.15 (m, 2H), 2.07 (m, 1H), 2.02–2.01 (m, 12H, 4 CH_3_), 1.23 (t, *J* = 6.8 Hz, 3H, 1 CH_3_); ^13^C-NMR (CDCl_3_): δ 183.25, 171.89, 171.23, 170.82, 170.03, 169.74, 135.83, 129.44, 128.51, 127.14, 82.52, 73.35, 72.87, 70.51, 68.23, 61.78, 61.71, 58.10, 37.46, 20.75, 20.69, 20.61, 20.59, 14.08; The NMR of **5a** is identical to that found in [65]; MS (ESI) *m*/*z* (%): 605.1 [M + Na]^+^ (100); HRMS (ESI, *m*/*z*) Calcd for C_26_H_35_N_2_O_11_S [M + H]^+^: 583.1962, Found: 583.1954.

*Ethyl (2,3,4,6-tetra-O-acetyl-β-d-glucopyranosyl)carbamothioyl-d-phenyl alaninate* (**5b**). Light yellow solid (2.59 g, 89%), m.p. 165.7–168.3 °C; *R*_f_ (*n*-hexane/EtOAc = 5:1): 0.45. [α${]}_{D}^{25}$ − 19.2 (*c* 0.1, CHCl_3_); ^1^H-NMR (CDCl_3_): δ 7.28–7.27 (m, 2H), 7.04 (m, 2H), 6.88–6.86 (m, 2H), 5.69 (br, 1H), 5.36 (t, *J* = 9.2 Hz, 2H), 5.09 (t, *J* = 9.2 Hz, 1H), 4.93 (t, *J* = 9.2 Hz, 1H), 4.30–4.28 (m, 1H), 4.17 (t, *J* = 6.8 Hz, 2H), 4.04 (d, *J* = 12.4 Hz, 1H), 3.87–3.84 (m, 1H), 3.44–3.40 (dd, *J*_1_ = 7.6 Hz, *J*_2_ = 5.6 Hz, 1H), 3.18 (d, *J* = 13.6 Hz, 1H), 2.05–2.01 (m, 12H, 4 CH_3_), 1.89–1.85 (m, 1H), 1.27 (t, *J* = 6.8 Hz, 3H, 1 CH_3_); ^13^C-NMR (CDCl_3_): δ 182.67, 171.54, 171.46, 170.74, 169.98, 169.69, 135.70, 129.50, 128.42, 127.17, 82.41, 73.32, 72.73, 70.65, 68.12, 61.87, 61.66, 57.86, 37.37, 20.73, 20.66, 20.62, 20.59, 14.15; MS (ESI) *m*/*z* (%): 605.1 [M + Na]^+^ (100); HRMS (ESI, *m*/*z*) Calcd for C_26_H_35_N_2_O_11_S [M + H]^+^: 583.1962, Found: 583.1957.

*(2R)-((2,3,4,6-tetra-O-Acetyl-β-d-glucopyranosyl)carbamothioylamino)-3-phenyl-1-propanol* (**5d**). Light yellow solid (2.45 g, 91%), m.p. 150.5–152.3 °C; *R*_f_ (*n*-hexane/EtOAc = 5:1): 0.40; [α${]}_{D}^{25}$ + 28.2 (*c* 0.5, CHCl_3_); ^1^H-NMR (CDCl_3_): δ 7.28–7.24 (m, 5H), 6.98–6.93 (m, 2H), 5.83 (br, 1H), 5.40–5.35 (m, 1H), 5.19 (m, 1H), 5.05 (m, 1H), 4.76 (br, 1H), 4.41–4.39 (m, 1H), 4.23–4.40 (m, 1H), 3.90–3.88 (m, 1H), 3.70–3.68 (m, 1H), 3.53–3.51 (m, 1H), 2.95–2.84 (m, 2H, CH_2_), 2.05 (m, 13 H, 4 CH_3_ + 1H); ^13^C-NMR (CDCl_3_): δ 183.08, 171.28, 170.90, 169.95, 16979, 137.57, 129.30, 128.59, 126.65, 82.91, 73.78, 72.88, 70.65, 68.23, 61.56, 57.10, 36.78, 20.79, 20.63; MS (ESI): *m*/*z* (%): 563.1 [M + Na]^+^ (100); HRMS (ESI, *m*/*z*) Calcd for C_24_H_33_N_2_O_10_S [M + H]^+^: 541.1856, Found: 541.1848.

*N-(2,3,4,6-tetra-O-Acetyl-β-d-glucopyranosyl)-N′-1-(S-)-phenylethyl thiourea* (**5e**). Light yellow solid (2.27 g, 89%), m.p. 153.3–154.7 °C; *R*_f_ (*n*-hexane/EtOAc = 5:1): 0.45; [α${]}_{D}^{25}$ + 4.0 (*c* 0.25, CHCl_3_); ^1^H-NMR (CDCl_3_): δ 7.37–7.32 (m, 5H), 6.66–6.35 (br, 1H), 5.66 (m, 1H), 5.37–5.32 (q, *J* = 9.2 Hz, 1H), 5.13–5.03 (m, 1H), 4.94–4.89 (m, 1H), 4.29–4.28 (m, 1H), 4.09–4.06 (d, *J* = 12.0 Hz, 1H), 3.83–3.82 (m, 1H), 2.11 (m, 2H), 2.04 (m, 12H, 4 CH_3_), 1.53 (d, *J* = 6.4 Hz, 3H, CH_3_); ^13^C-NMR (CDCl_3_): δ 182.45, 171.62, 170.74, 169.86, 169.73, 129.01, 127.85, 126.01, 90.17, 82.92, 73.28, 72.62, 70.89, 68.33, 67.67, 53.90, 20.83, 20.76, 20.63, 20.61; MS (ESI): *m*/*z* (%): 533.1 [M + Na]^+^ (100); HRMS (ESI, *m*/*z*) Calcd for C_23_H_31_N_2_O_9_S [M + H]^+^: 511.1750, Found: 511.1743.

*N-(2,3,4,6-tetra-O-Acetyl-β-d-glucopyranosyl)-N′-1-(R-)-phenylethyl thiourea* (**5f**). Light yellow solid (2.45 g, 95%), m.p. 79.5–82.0 °C; *R*_f_ (*n*-hexane/EtOAc = 5:1): 0.45; [α${]}_{D}^{25}$ + 4.8 (*c* 0.5, CHCl_3_); ^1^H-NMR (CDCl_3_): δ 7.35–7.29 (m, 5H), 6.96 (br, 2H), 5.71 (br, 1H), 5.35–5.27 (q, *J* = 9.6 Hz, 1H), 5.05 (t, *J* = 9.6 Hz, 1H), 4.92 (m, 1H), 4.35–4.31 (m, 1H), 4.09–4.03 (m, 1H), 3.84–3.82 (m, 1H), 2.04–1.99 (m, 13H, 4 CH_3_ + 1H), 1.50 (d, *J* = 5.6 Hz, 3H, CH_3_); ^13^C-NMR (CDCl_3_): δ 182.45, 171.48, 170.83, 169.91, 169.76, 128.95, 127.78, 126.04, 82.86, 73.37, 72.87, 70.91, 68.37, 68.32, 61.71, 53.85, 20.78, 20.76, 20.62, 20.56; MS (ESI): *m*/*z* (%): 533.1 [M + Na]^+^ (100); HRMS (ESI, *m*/*z*) Calcd for C_23_H_31_N_2_O_9_S [M + H]^+^: 511.1750, Found: 511.1752.

*N-(2,3,4,6-tetra-O-Acetyl-β-d-glucopyranosyl)-N′-benzyl thiourea* (**5g**). Light yellow solid (2.37 g, 96%), m.p. 72.7–74.3 °C; *R*_f_ (*n*-hexane/EtOAc = 5:1): 0.50; [α${]}_{D}^{25}$ + 5.2 (*c* 0.25, CHCl_3_); ^1^H-NMR (CDCl_3_): δ 7.37–7.28 (m, 5H), 6.62 (br, 2H), 5.73 (t, *J* = 8.4 Hz, 1H), 5.37 (t, *J* = 10.0 Hz, 1H), 5.09 (t, *J* = 9.6 Hz, 1H), 4.99 (t, *J* = 9.6 Hz, 1H), 4.77 (br, 1H), 4.36–4.32 (dd, *J*_1_ = 7.6 Hz, *J*_2_ = 4.8 Hz, 1H), 4.05 (d, *J* = 12.4 Hz, 1H), 3.88–3.84 (dd, *J*_1_ = 5.2 Hz, *J*_2_ = 4.4 Hz, 1H), 2.10–2.05 (m, 13H, 4 CH_3_ + 1 H); ^13^C-NMR (CDCl_3_): δ 170.85, 169.93, 169.74, 128.93, 127.94, 82.96, 73.49, 72.74, 70.82, 68.35, 61.70, 20.79, 20.72, 20.64, 20.62; MS (ESI): *m*/*z* (%): 519.1 [M + Na]^+^ (100); HRMS (ESI, *m*/*z*) Calcd for C_22_H_29_N_2_O_9_S [M + H]^+^: 497.1594, Found: 497.1594.

*N-(2,3,4,6-tetra-O-Acetyl-β-d-glucopyranosyl)-N′-benzamido thiourea* (**5h**). Light yellow solid (2.51 g, 95%), m.p. 125.6–127.2 °C; *R*_f_ (*n*-hexane/EtOAc = 5:1): 0.40; [α${]}_{D}^{25}$ + 6.2 (*c* 0.28, CHCl_3_); ^1^H-NMR (CDCl_3_): δ 7.92–7.90 (d, *J* = 7.6 Hz, 2H), 7.63 (m, 1H), 7.54 (m, 2H), 5.75 (t, *J* = 8.8 Hz, 1H), 5.37 (t, *J* = 9.6 Hz, 1H), 5.06 (m, 2H), 4.30 (d, *J* = 10.0 Hz, 1H), 4.03 (br, 1H), 3.86 (d, *J* = 8.0 Hz, 1H), 2.06–2.00 (m, 13H, 4 CH_3_ + 1H), 1.95 (br, 2H); ^13^C-NMR (CDCl_3_): δ 170.90 169.87, 169.64, 132.99, 130.92, 128.97, 127.48, 82.70, 73.73, 72.60, 70.40, 68.18, 61.53, 20.78, 20.61, 20.57; MS (ESI): *m*/*z* (%): 548.0 [M + Na]^+^ (100); HRMS (ESI, *m*/*z*) Calcd for C_22_H_28_N_3_O_10_S [M + H]^+^: 526.1495, Found: 526.1489. 

*N-(2,3,4,6-tetra-O-Acetyl-b-d-glucopyranosyl)-N′-deoxyquininyl thiourea* (**5i**) [66]. Light yellow solid (2.86 g, 81%), m.p. 94.2–95.7 °C, *R*_f_ (*n*-hexane/EtOAc = 3:1): 0.40. [α${]}_{D}^{25}$ − 67.2 (*c* 0.25, CHCl_3_); Lit. [66] white solid, m.p. 139–140 °C, [α${]}_{D}^{25}$ = −70.87° (*c* = 1.0, CHCl_3_); ^1^H-NMR (CDCl_3_): δ 8.74–8.73 (d, *J* = 4.0 Hz, 1H), 8.04–8.02 (d, *J* = 8.8 Hz, 1H), 7.72 (br, 1H), 7.41–7.29 (m, 3H), 5.71–5.66 (m, 2H), 5.33 (t, *J* = 9.2 Hz, 1H), 5.32 (m, 1H), 5.11–4.98 (m, 4H), 4.30 (d, *J*_1_ = 8.4 Hz, *J*_2_ = 3.6 Hz, 1H), 4.11 (br, 1H), 4.02 (s, 3H, OCH_3_), 3.82 (d, *J* = 8.4 Hz, 1H), 3.26–3.23 (m, 4H), 2.81 (br, 2H), 2.35 (br, 1H), 2.03–2.01 (m, 12H, 4 CH_3_), 1.71 (m, 3H), 1.37–1.27 (m, 2H), 0.96–0.93 (m, 1H). ^13^C-NMR (CDCl_3_): δ 184.03, 181.13, 170.63, 169.89, 169.62, 158. 15, 147.62, 144.76, 140.31, 131.82, 130.94, 128.84, 121.99, 115.27, 73.32, 72.92, 70.80, 68.19, 65.58, 61.58, 55.79, 40.82, 38.90, 30.56, 27.17, 25.34, 24.12, 20.74, 20.69, 20.60, 19.18, 13.74; the NMR of **5i** is identical to the published data [66]; MS (ESI): *m*/*z* (%): 735.1 [M + Na]^+^, 713.2, 701.4, 588.3 (100).]; HRMS (ESI, *m*/*z*) Calcd for C_35_H_45_N_4_O_10_S [M + Na]^+^: 713.2856, Found: 713.2863.

*Methyl* *(2,3,4,6-tetra-O-acetyl-b-d-glucopyranosyl)carbamothioylamino-(2S-hydroxyl-3R-phenyl)-1-propanate* (**5j**). Light yellow solid (2.48 g, 85%), m.p. 135.9–137.5 °C; *R*_f_ (*n*-hexane/EtOAc = 3:1): 0.40; [α${]}_{D}^{25}$ − 10.6 (*c* 0.4, CHCl_3_); ^1^H-NMR (CDCl_3_): δ 7.56–7.54 (m, 1H), 7.38–7.30 (m, 4H), 7.05–7.03 (m, 1H), 6.27–6.26 (m, 1H), 5.85 (br, 1H), 5.35 (t, *J* = 9.2 Hz, 1H), 5.11 (t, *J* = 9.6 Hz, 2H), 4.62 (s, 1H), 4.35–4.31 (m, 1H), 4.15–4.09 (m, 1H), 3.87–3.85 (m, 1H), 3.80 (s, 3H, OCH_3_), 2.09 (m, 2H), 2.06–2.03 (m, 12H, 4 CH_3_). ^13^C-NMR (CDCl_3_): δ 183.79, 173.65, 171.72, 170.82, 170.03, 169.65, 138.20, 128.70, 127.90, 126.91, 90.14, 82.81, 73.53, 72.92, 70.80, 68.22, 61.74, 59.32, 52.98, 20.72, 20.64, 20.62; MS (ESI): *m*/*z* (%): 607.1 [M + Na]^+^ (100); HRMS (ESI, *m*/*z*) Calcd. for C_25_H_33_N_2_O_12_S [M + H]^+^: 585.1754, Found: 585.1751. 

### 3.3. Biological Assay

All bioassays were performed on representative test organisms reared in the laboratory. The bioassay was repeated at 25 ± 1 °C according to statistical requirements. 

#### 3.3.1. Larvicidal Activity against *Mythimna separata* Walker

The larvicidal activity of the title compounds and contrast with control group (CG) against oriental armyworm (*Mythimna separata* Walker) was tested according to the leaf-dip method using the reported procedure [14]. Leaf disks (about 5 cm) were cut from fresh corn leaves and then were dipped into the test solution for 3−5 s. After air drying, the treated leaf disks were placed individually into a glass-surface vessel (9 cm). Each dried treated leaf disk was infested with 10 third instar oriental armyworm larvae. Percentage mortalities were evaluated 4 days after treatment. Leaves treated with acetone were provided as controls (CG). Each treatment was performed three times. The assessments of larvicidal activity of **5a**–**j** were made on a dead/alive basis, and mortality rates were corrected using Abbott’s formula. Evaluations were based on a percentage scale of 0−100, in which 0 = no activity and 100 = total kill. The insecticidal activity is summarized in Table 1.

#### 3.3.2. Growth Inhibitory Activities to *Mythimna separata* Walker

The growth inhibitory activity of the compounds **5a**–**c** and contrast with control group (CG) was tested according to the leaf-dip method using the above procedure. The inhibitory rates of the compounds, summarized in Table 2, were calculated according to the following formula:

$$\text{Inhibitory rate}\;\left(\text{\%}\right)\;=\;\frac{\text{average increased weight of control - average increased weight of test}}{\text{average increased weight of control}}\;\times \;\text{100\%}$$

## 4. Conclusions 

In summary, a series of novel glucosylthioureas with “anti-chitin structure” have been prepared through four steps from d-glucose and various amines, and this synthetic route is notable for its convenience and high efficiency. The larvicidal activities of these compounds on *Mythiman separata* Walker have been also investigated. Although no obvious larvicidal ability was found for any of these thioureas, some of them show moderate growth inhibitory activities (up to 47.38%) on *Mythiman separata* Walker. Our attempt to use natural materials such as carbohydrates and amino acids as sources for synthesis of new pesticides may open a new window for the development of green pesticides.

## Figures and Tables

**Table 1:** The larvicidal activities against *Mythimna separata* Walker of **5a**–**j**.

Larvicidal Activity (%) ^a^ at (mg/L)						
Compound	6.25	12.5	25.0	50.0	100.0	CG ^b^
**5a**	13.3	10.0	13.3	16.7	10.0	13.3
**5b**	0	10.0	10.0	10.0	16.7	13.3
**5c**	3.3	3.3	6.7	3.3	13.3	13.3
**5d**	0	6.7	3.3	3.3	13.3	0
**5e**	0	3.3	0	10.0	0	0
**5f**	0	0	3.3	0	0	3.3
**5g**	0	3.3	0	0	0	3.3
**5h**	3.3	3.3	0	0	3.3	3.3
**5i**	0	6.7	0	3.3	3.3	0
**5j**	0	3.3	0	6.7	0	0

^a^ The compound tested was dissolved in acetone at the indicated concentration; ^b^ CG = Control group.

**Table 2:** The growth inhibitory activities to *Mythimna separata* Walker of **5a**–**c**.

Growth Inhibition Activity (%) ^a^ at (mg/L)						
Compds	6.25	12.5	25.0	50.0	100.0	CG ^b^
**5a**	6.07	4.59	16.56	30.16	39.34	-
**5b**	1.97	8.85	6.89	26.56	34.75	-
**5c**	7.87	9.67	25.25	35.25	47.38	-

^a^ The compound tested was dissolved in acetone at the indicated concentration. ^b^ CG = Control group.

## Supplementary Materials

The following supplementary data (^1^H-NMR, ^13^C-NMR, and HRMS spectra) associated with this article are available online at http://www.mdpi.com/1420-3049/21/7/925/s1.

## References

[1] Heckel D.G. (2012). Insecticide resistance after *Silent Spring*. Science.

[2] Suzuki H., Tomizawa M., Ito Y., Abe K., Noro Y., Kamijima M. (2013). A potential target for organophosphate insecticides leading to spermatotoxicity. J. Agric. Food Chem..

[3] Storm J.E. (2012). Patty's Toxicology.

[4] Matthews G.A., Bateman R., Miller P. (2014). Safety precautions. Pesticide Application Methods.

[5] Casida J.E. (2011). Curious about pesticide action. J. Agric. Food Chem..

[6] Alexander M. (1973). Nonbiodegradable and other racalcitrant molecules. Biotechnol. Bioeng..

[7] Maguire R.J., Carey J.H., Hart J.H., Tkacz R.J., Lee H.B. (1989). Persistence and fate of deltamethrin sprayed on a pond. J. Agric. Food Chem..

[8] Weston D.P., Lydy M.J. (2014). Toxicity of the insecticide fipronil and is degradates to benthic macroinvertebrates of urban streams. Environ. Sci. Technol..

[9] Dawkar V.V., Chikate Y.R., Lomate P.R., Dholakia B.B., Gupta V.S., Giri A.P. (2013). Molecular insights into resistance mechanisms of lepidopteran insect pests against toxicants. J. Proteome Res..

[10] Cabras P., Angioni A. (2000). Pesticide residues in grapes, wine, and their processing products. J. Agric. Food Chem..

[11] Jeschke P., Nauen R., Schindler M., Elbert A. (2011). Overview of the status and global strategy for neonicotinoids. J. Agric. Food Chem..

[12] Mori K., Ohkawa H., Miyagawa H., Lee P.W. (2007). Pesticide Chemistry: Crop Protection, Public Health, Environmental Safety.

[13] Ning J., Kong F.Z., Lin B.M., Lei H.D. (2003). Large-scale preparation of the phytoalexin elicitor glucohexatose and its application as a green pesticide. J. Agric. Food Chem..

[14] Sinthusiri J., Soonwera M., Boonmeesupmak P. (2013). Green insecticide from herbal essential oils against house fly, *Musca domestica* L. (Muscidae: Diptera). J. Agric. Tech..

[15] Qian X.H., Lee P.W., Cao S. (2010). China: Forward to the green pesticides via a basic research program. J. Agric. Food Chem..

[16] Kanokmedhakul S., Kanokmedhakul K., Prajuabsuk T., Panichajakul S., Panyamee P., Prabpai S., Kongsaeree P. (2005). Azadirachtin derivatives from seed kernels of Azadirachta excels. J. Nat. Prod..

[17] Cantrell C.L., Dayan F.E., Duke S.O. (2012). Natural products as sources for new pesticides. J. Nat. Prod..

[18] Wiwattanapatapee R., Sae-Yun A., Petcharat J., Ovatlarnporn C., Itharat A. (2009). Development and evaluation of granule and emulsifiable concentrate formulations containing derris elliptica extract for crop pest control. J. Agric. Food Chem..

[19] Feyereisen R. (2015). Insect P450 inhibitors and insecticides: Challenges and opportunities. Pest. Manag. Sci..

[20] Petroski R.J., Stanley D.W. (2009). Natural compounds for pest and weed control. J. Agric. Food Chem..

[21] Cohen E. (2001). Chitin synthesis and inhibition: A revisit. Pest. Manag. Sci..

[22] Merzendorfer H. (2013). Chitin synthesis inhibitors: Old molecules and new developments. Insect Sci..

[23] Hayashi N., Sasama Y., Takahashi N., Ikemi N. (2013). Cyflumetofen, a novel acaricide—Its mode of action and selectivity. Pest. Manag. Sci..

[24] Sun R.F., Lü M.Y., Chen L., Li Q.S., Song H.B., Bi F.C., Huang R.Q., Wang Q.M. (2008). Design, synthesis, bioactivity, and structure-activity relationship (SAR) studies of novel benzoylphenylureas containing oxime ether group. J. Agric. Food Chem..

[25] DeMilo A.B., Ostromecky D.M., Chang S.C., Redfern R.E., Fye R.L. (1978). Heterocyclic analogs of diflubenzuron as growth and reproduction inhibitors of the fall armyworm and house fly. J. Agric. Food Chem..

[26] Sun R.F., Wang Z.W., Li Y.Q., Xiong L.X., Liu Y.X., Wang Q.M. (2013). Design, synthesis, and insecticidal evaluation of new benzoylureas containing amide and sulfonate groups based on the sulfonylurea receptor protein binding site for diflubenzuron and glibenclamide. J. Agric. Food Chem..

[27] Ascher K.R.S., Nemny N.E. (1976). Toxicity of the chitin synthesis inhibitors, diflubenzuron and its dichloro-analogue, to *Spodoptera littoralis* larvae. Pestic. Sci..

[28] Zhang J., Tang X.H., Ishaaya I., Cao S., Wu J.J., Yu J.L., Li H., Qian X.H. (2010). Synthesis and insecticidal activity of heptafluoroisopropyl-containing benzoylphenylurea structures. J. Agric. Food Chem..

[29] Rama F., Meazza G., Bettarini F., Piccardi P., Massardo P., Caprioli V. (1992). Synthesis and bioactivity of some fluorine-containing benzoyl arylureas. Part II: Insecticidal products in which the aryl group bears a polyfluoroalkoxy or (polyfluoroalkoxy)alkoxy side chain. Pestic. Sci..

[30] Sreelatha T., Hymavathi A., Babu K.S., Murthy J.M., Pathipati U.R., Rao J.M. (2009). Synthesis and insect antifeedant activity of plumbagin derivatives with the amino acid moiety. J. Agric. Food Chem..

[31] Pittendrigh B.R., Laskowski H., O’Shea G., Larsen A., Wolfe R. (2001). Carbohydrate-based mosquito control: A field test of the concept. Environ. Entomol..

[32] Steve P., Jan T., Perveen F. (2012). Insecticides-Advances in Integrated Pest Management.

[33] Bland J.M., Edwards J.V., Eaton S.R., Lax A.R. (1993). Potential of natural peptidic compounds as leads for novel pesticides. Pestic. Sci..

[34] Mao M.Z., Li Y.X., Zhou Y.Y., Zhang X.L., Liu Q.X., Di F.J., Song H.B., Xiong L.X., Li Y.Q., Li Z.M. (2014). Synthesis and insecticidal evaluation of novel *N*-pyridylpyrazolecarboxamides containing an amino acid methyl ester and their analogues. J. Agric. Food Chem..

[35] Berecibar A., Grandjean C., Siriwardena A. (1999). Synthesis and biological activity of natural aminocyclopentitol glycosidase inhibitors: Mannostatins, Trehazolin, Allosamidins, and their analogues. Chem. Rev..

[36] Shiozaki M., Kobayashi Y., Arai M., Haruyama H. (1994). Synthesis of 6-epi-trehazolin from d-ribonolactone: Evidence for the non-existence of a 5,6-ringfused structural isomer of 6-epi-trehazolin. Tetrahedron Lett..

[37] Kobayashi Y., Shiozaki M., Ando O. (1995). Syntheses of Trehazolin derivatives and evaluation as glycosidase inhibitors. J. Org. Chem..

[38] Elshahawi S.I., Shaaban K.A., Kharel M.K., Thorson J.S. (2015). A comprehensive review of glycosylated bacterial natural products. Chem. Soc. Rev..

[39] Yamagishi T., Uchida C., Ogawa S. (1995). Total synthesis of the Trehalase inhibitor Salbostatin. Chem. Eur. J..

[40] Vertesy L., Fehlhaber H.W., Schulz A. (1992). Glycosidase Inhibitor Salbostatin, process for Its Preparation, and Its Use. US Patent.

[41] Trapero A., Egido-Gabás M., Bujons J., Llebaria A. (2015). Synthesis and evaluation of hydroxymethylaminocyclitols as glycosidase inhibitors. J. Org. Chem..

[42] Shing T.K.M., Cheng H.M. (2008). Intramolecular direct aldol reactions of sugar diketones: Syntheses of valiolamine and validoxylamine. Org. Lett..

[43] Fukase H., Horii S. (1992). Synthesis of valiolamine and its *N*-substituted derivatives AO-128, validoxylamine G, and validamycin G via branched-chain inosose derivatives. J. Org. Chem..

[44] Cipolla L., Sgambato A., Forcella M., Fusi P., Parenti P., Cardona F., Bini D. (2014). *N*-Bridged 1-deoxynojirimycin dimers as selective insect trehalase inhibitors. Carbohydr. Res..

[45] Schareina T., Zapf A., Cotté A., Müller N., Beller M. (2008). A practical and improved Copper-catalyzed synthesis of the central intermediate of diafenthiuron and related products. Org. Process Res. Dev..

[46] Knox J.R., Toia R.F., Casida J.E. (1992). Insecticidal thioureas: Preparation of [phenoxy-4–3H]diafenthiuron, the corresponding carbodiimide, and related compounds. J. Agric. Food Chem..

[47] Pascual A., Rindlisbacher A., Schmidli H., Stamm E. (1995). *N*-(pyrid-3-yl)thioureas and derivatives as acaricides. II. Quantitative structure-activity relationships and chemodynamic behaviour. Pestic. Sci..

[48] Pascual A., Rindlisbacher A. (1994). *N*-(pyrid-3-yl)thioureas and derivatives as acaricides. I. Synthesis and biological properties. Pestic. Sci..

[49] Mao C.H., Wang Q.M., Huang R.Q., Bi F.C., Chen L., Liu Y.X., Shang J. (2004). Synthesis and insecticidal evaluation of novel *N*-oxalyl derivatives of tebufenozide. J. Agric. Food Chem..

[50] Wang H., Yang Z.K., Fan Z.J., Wu Q.J., Zhang Y.J., Mi N., Wang S.X., Zhang Z.C., Song H.B., Liu F. (2011). Synthesis and insecticidal activity of *N*-tert-butyl-*N*,*N*′-diacylhydrazines containing 1,2,3-thiadiazoles. J. Agric. Food Chem..

[51] Akamatsu M. (2011). Importance of physicochemical properties for the design of new pesticides. J. Agric. Food Chem..

[52] Morou E., Lirakis M., Pavlidi N., Zotti M., Nakagawa Y., Smagghe G., Vontas J., Swevers L. (2013). A new dibenzoylhydrazine with insecticidal activity against *Anopheles mosquito* larvae. Pest. Manag. Sci..

[53] Sawada Y., Yanai T., Nakagawa H., Tsukamoto Y., Yokoi S., Yanagi M., Toya T., Sugizaki H., Kato Y., Shirakura H. (2003). Synthesis and insecticidal activity of benzoheterocyclic analogues of *N*′-benzoyl-*N*-(*tert*-butyl)benzohydrazide: Part 1. Design of benzoheterocyclic analogues. Pest. Manag. Sci..

[54] Zhou S., Jia Z.H., Xiong L.X., Yan T., Yang N., Wu G.P., Song H.B., Li Z.M. (2014). Chiral dicarboxamide scaffolds containing a sulfiliminyl moiety as potential ryanodine receptor activators. J. Agric. Food Chem..

[55] Fan L.L., Guo Y., Zhi X.Y., Yu X., Xu H. (2014). Stereoselective synthesis of 2α-chloropicropodophyllotoxins and insecticidal activity of their esters against oriental armyworm, Mythimna separata walker. J. Agric. Food Chem..

[56] Li J., Wang Z.Y., Wu Q.Y., Yang G.F. (2015). Design, synthesis and insecticidal activity of novel 1,1-dichloropropene derivatives. Pest. Manag. Sci..

[57] He S.Z., Shao Y.H., Fan L.L., Che Z.P., Xu H., Zhi X.Y., Wang J.J., Yao X.J., Qu H. (2013). Synthesis and quantitative structure–activity relationship (QSAR) study of novel 4-acyloxypodophyllotoxin derivatives modified in the A and C rings as insecticidal agents. J. Agric. Food Chem..

[58] Redemann C.E., Niemann C. (1942). Acetobromoglucose. Org. Synth..

[59] Liu K., Cui H.F., Nie J., Dong K.Y., Li X.J., Ma J.A. (2007). Highly enantioselective michael addition of aromatic ketones to nitroolefins promoted by chiral bifunctional primary amine-thiourea catalysts based on saccharides. Org. Lett..

[60] Gao P., Wang C.G., Wu Y., Zhou Z.H., Tang C.C. (2008). Eur. J. Org. Chem..

[61] Vakulya B., Varga S., Csámpai A., Soós T. (2005). Highly enantioselective conjugate addition of nitromethane to chalcones using bifunctional Cinchona organocatalysts. Org. Lett..

[62] Zhang H.L., Syed S., Barbas C.F. (2010). Highly enantio- and diastereoselective Mannich reactions of glycine Schiff bases with in situ generated *N*-Boc-imines catalyzed by a Cinchona alkaloid thiourea. Org. Lett..

[63] Gennari C., Carcano M., Donghi M., Mongelli N., Vanotti E., Vulpetti A. (1997). Taxol semisynthesis: A highly enantio- and diastereoselective synthesis of the side chain and a new method for ester formation at C-13 using thioesters. J. Org. Chem..

[64] Hu Z.Y., Erhardt P.W. (1997). Utilization of a benzoyl migration to effect an expeditious synthesis of the paclitaxel C-13 side chain. Org. Process Res. Dev..

[65] Li Y.X., Chen W., Yang X.P., Yu G.P., Mao M.Z., Zhou Y.Y., Liu T.W., Li Z.M. (2011). Regioselective synthesis of novel 3-Thiazolidine acetic acid derivatives from glycosido ureides. Chem. Biol. Drug Des..

[66] Kong S.S., Fan W.D., Wu G.P., Miao Z.W. (2012). Enantioselective synthesis of tertiary α-hydroxy phosphonates catalyzed by carbohydrate/cinchona alkaloid thiourea organocatalysts. Angew. Chem. Int. Ed..

