# Supplementary material for: A New Class of Glucosyl Thioureas: Synthesis and Larvicidal Activities
Source: Molecules. 2016 Jul 16;21(7):925. doi: 10.3390/molecules21070925 (PMC6274248; doi:10.3390/molecules21070925)
Supplement: Supplementary file 1 [file molecules-21-00925-s001.pdf]

Supplementary Materials: A New Class of Glucosyl Thioureas: Synthesis and Larvicidal Activities

Ping-An Wang [1,*,†], Jun-Tao Feng [2,†], Xing-Zi Wang [1] and Mu-Qiong Li [1]

Copies of NMR and MS of glucosyl thioureas 5a–5j

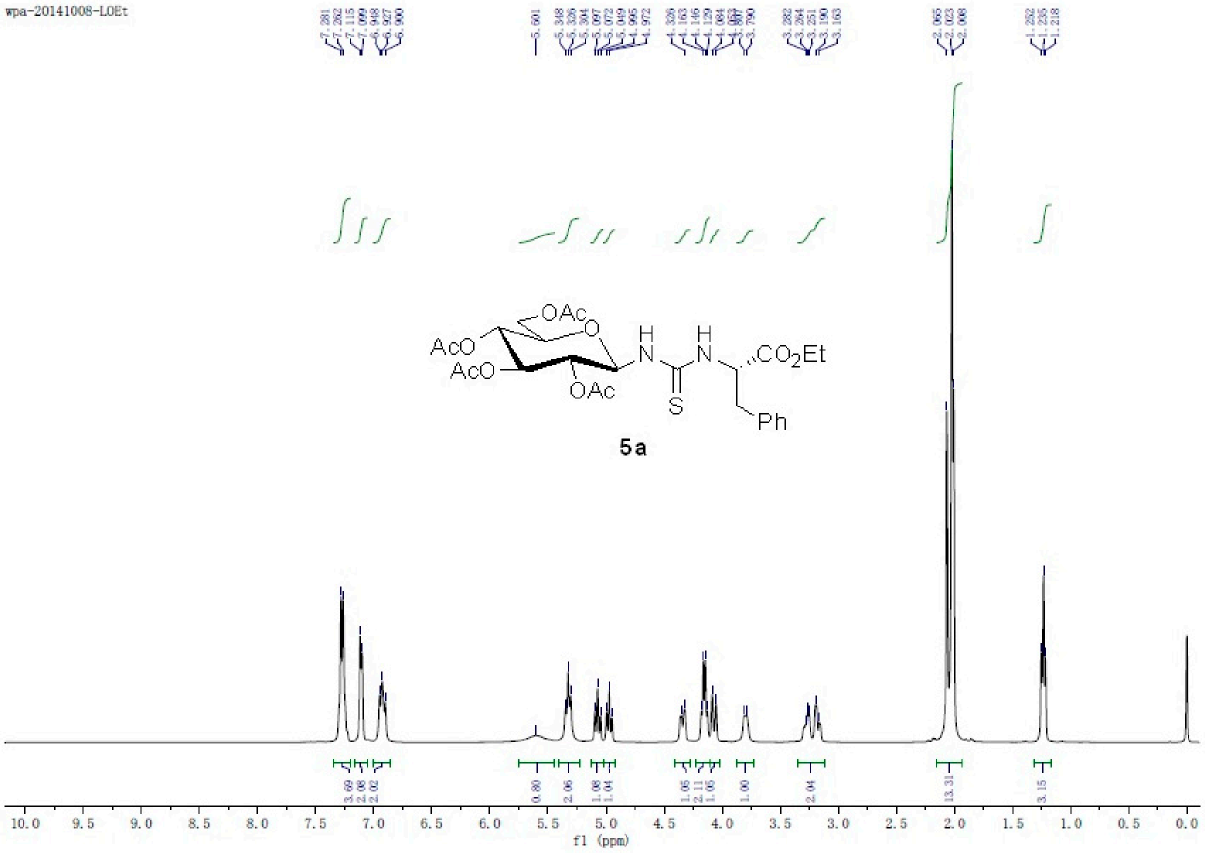

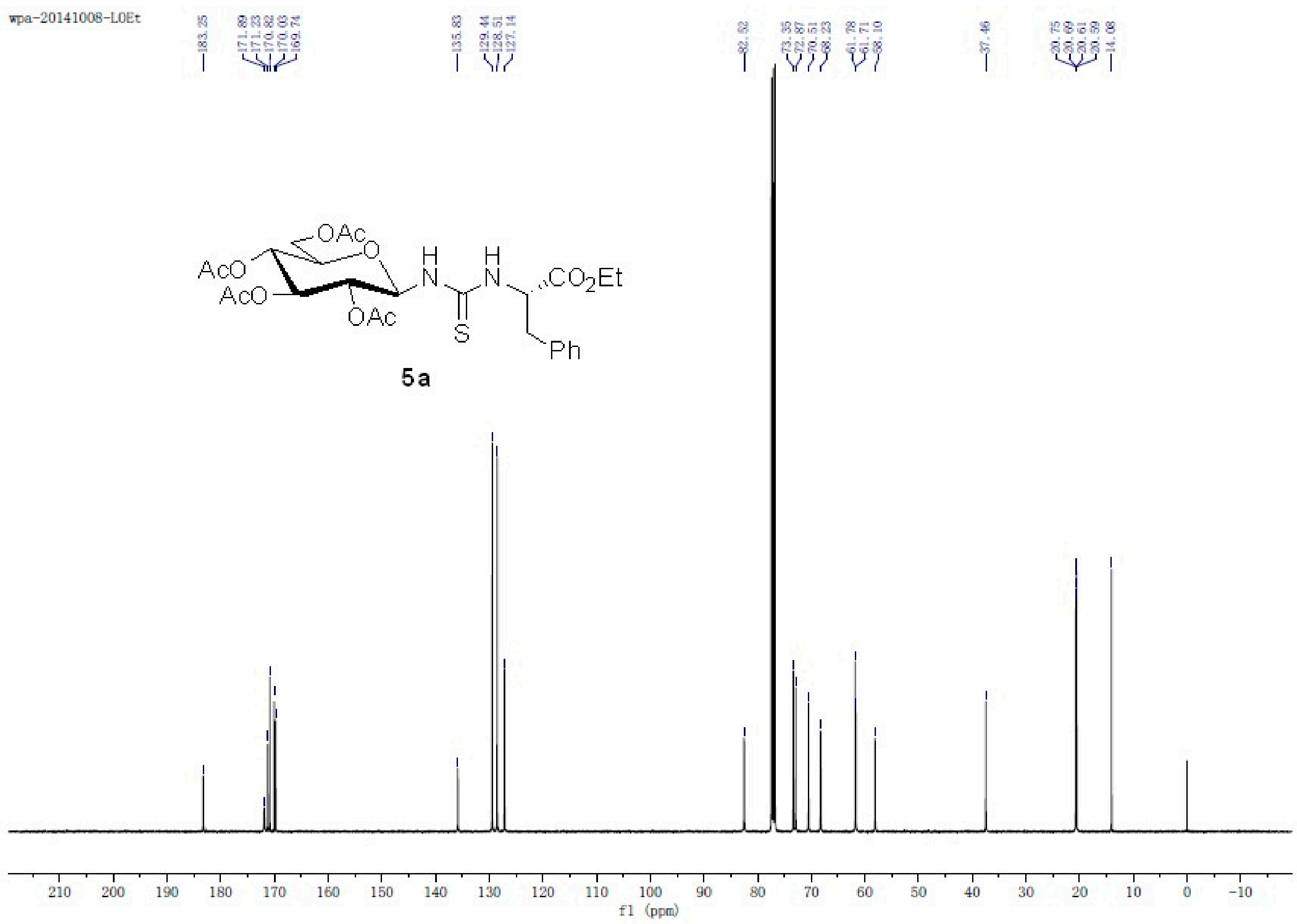
wpa-20141008-LOEt
183.25
171.89
171.23
170.82
170.05
169.74
135.83
129.44
128.51
127.14
82.52
73.35
72.87
70.51
68.23
61.78
61.71
58.10
37.46
20.75
20.69
20.61
20.59
14.08
5a
f1 (ppm)

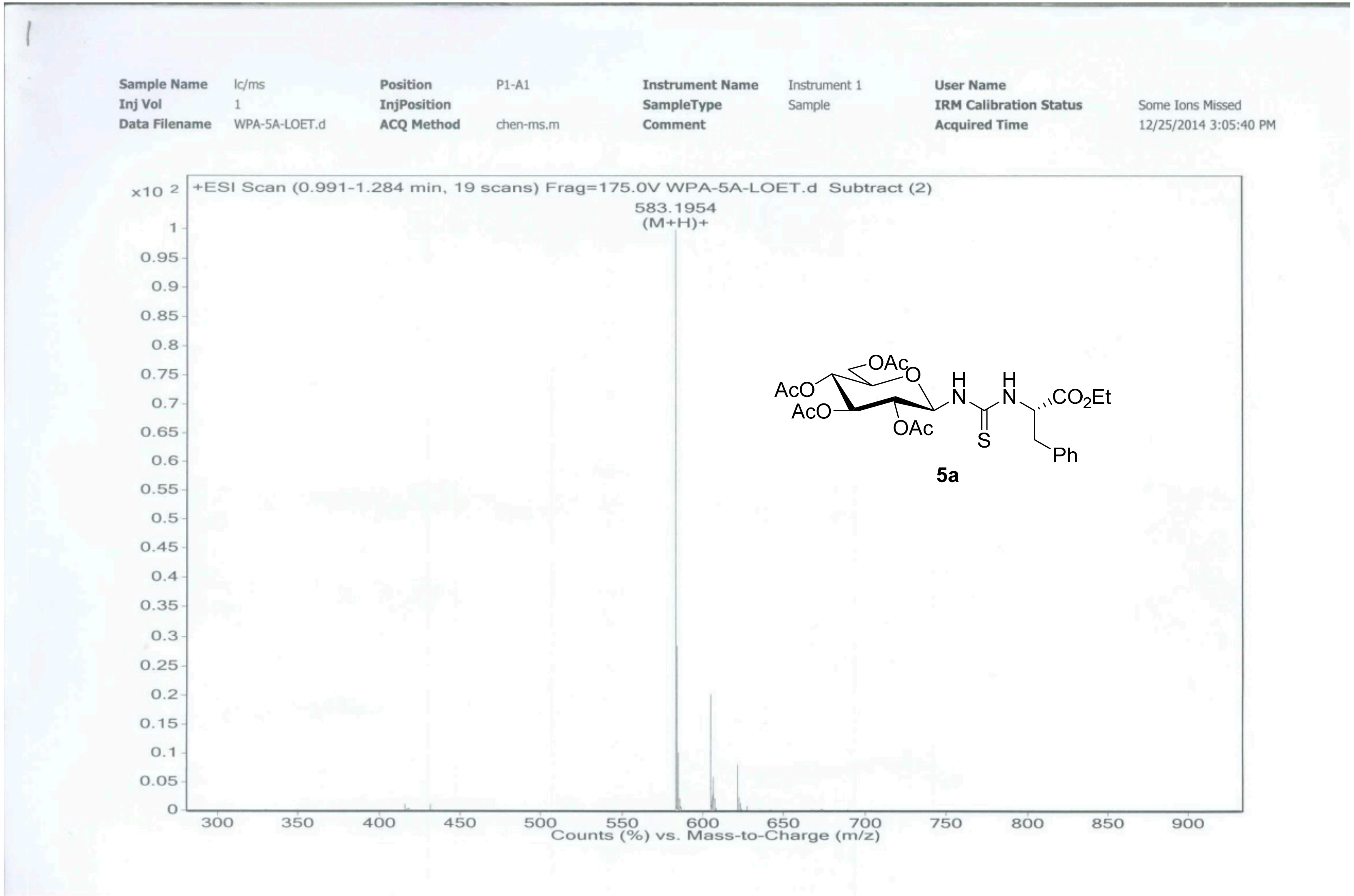

Figure S1. ^{1}H-NMR, ^{13}C-NMR and HRMS of **5a**.

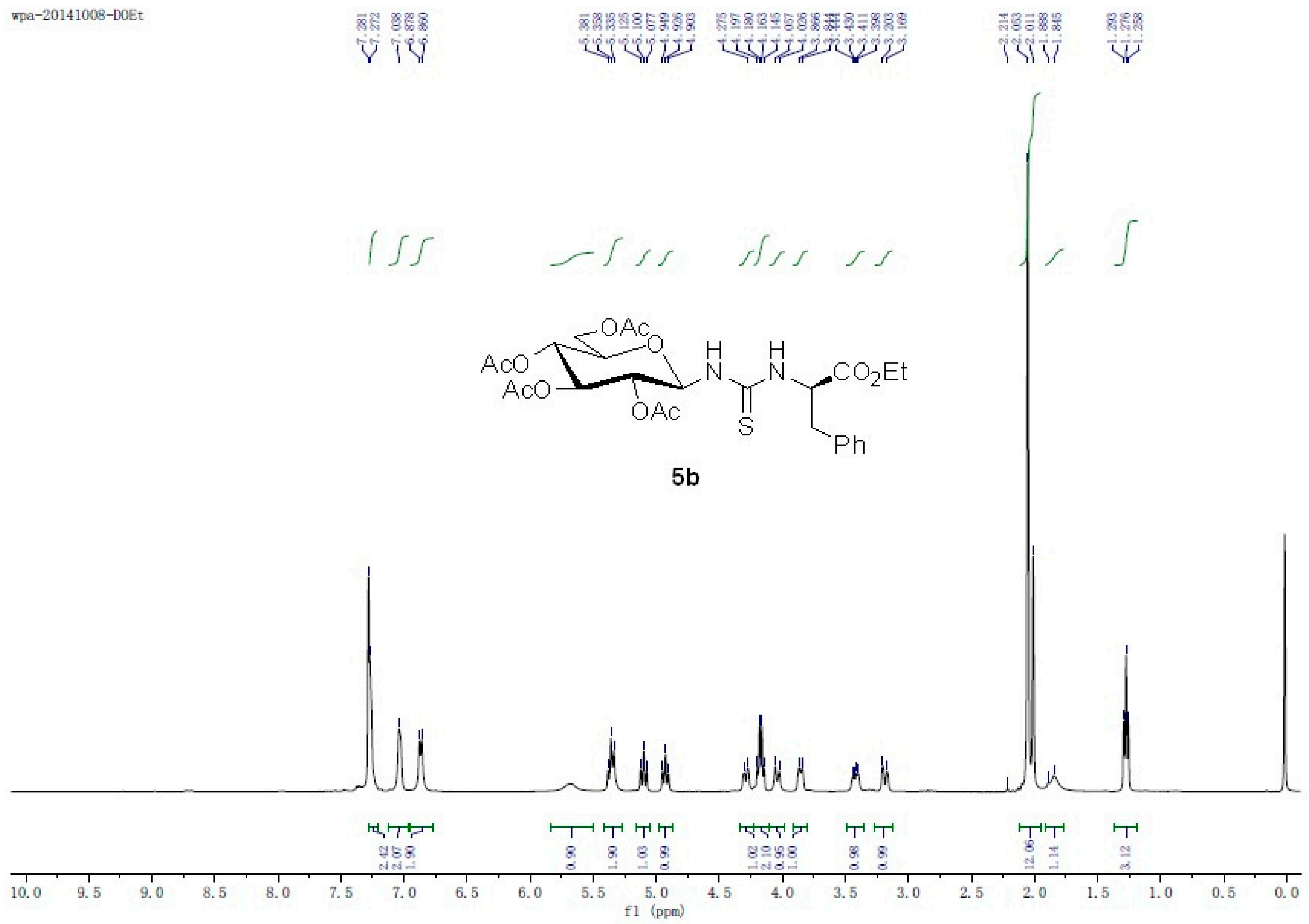
wpa-20141008-DOEt
AcO
AcO
OAc
OAc
H
N
H
N
CO2Et
S
Ph
5b
f1 (ppm)

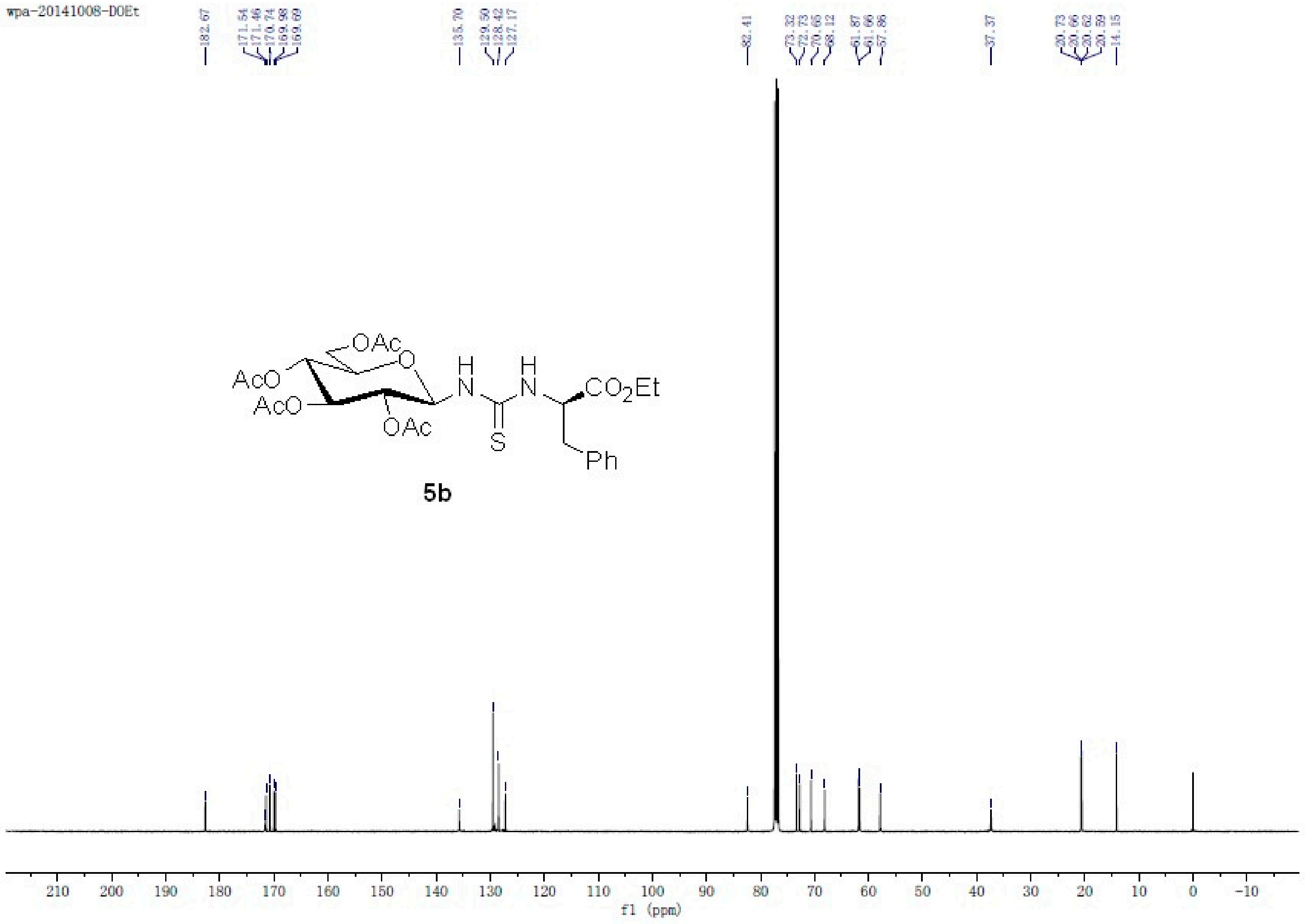
wpa-20141008-DOEt
182.67
171.54
171.46
170.74
169.98
169.69
135.70
129.50
128.42
127.17
82.41
73.32
72.73
70.65
68.12
61.87
61.66
57.86
37.37
20.73
20.66
20.62
20.59
14.15
AcO
AcO
OAc
OAc
O
H
N
H
N
S
CO_2Et
Ph
5b
f1 (ppm)

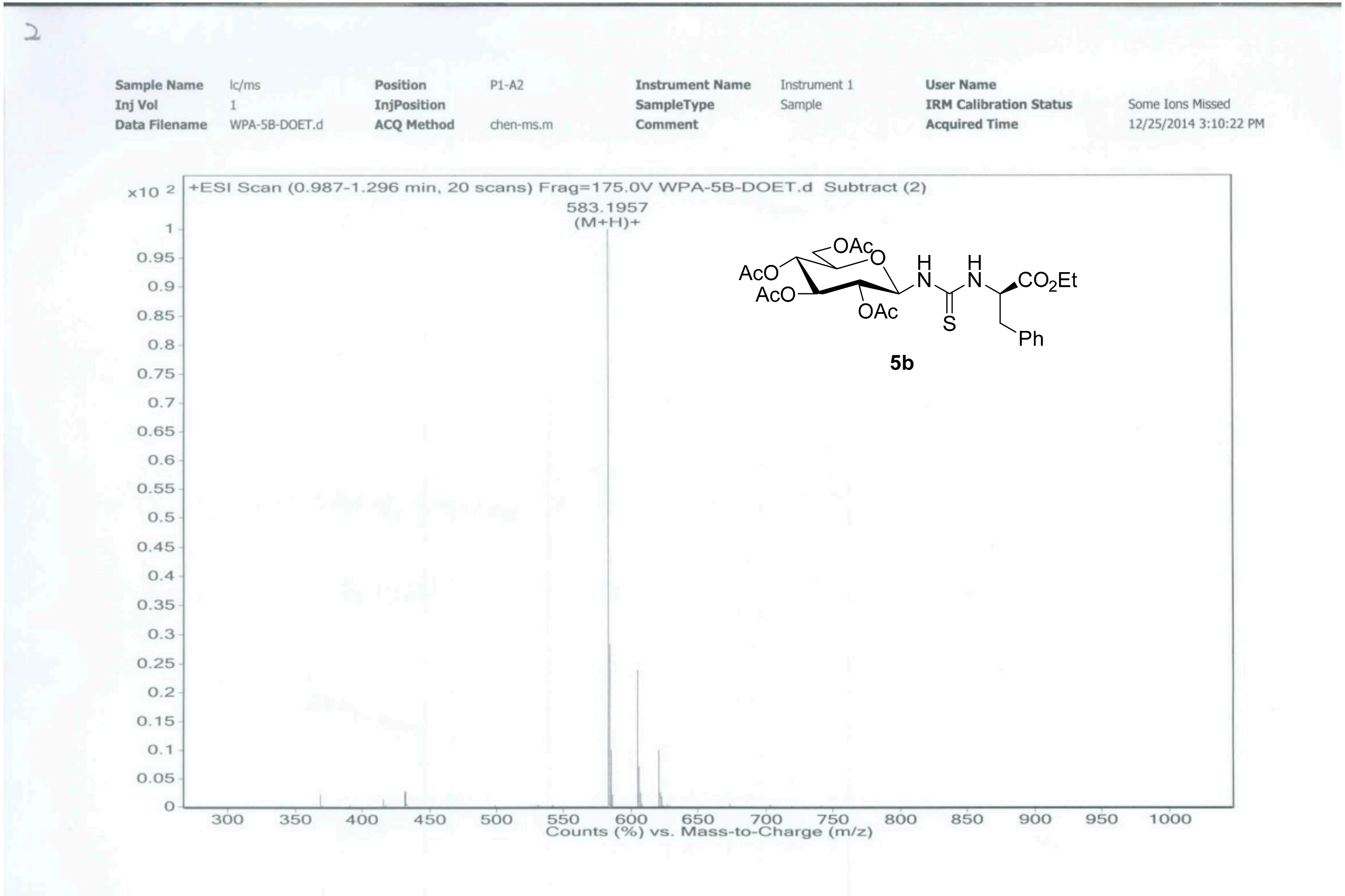

Figure S2. ^{1}H-NMR, ^{13}C-NMR and HRMS of **5b**.

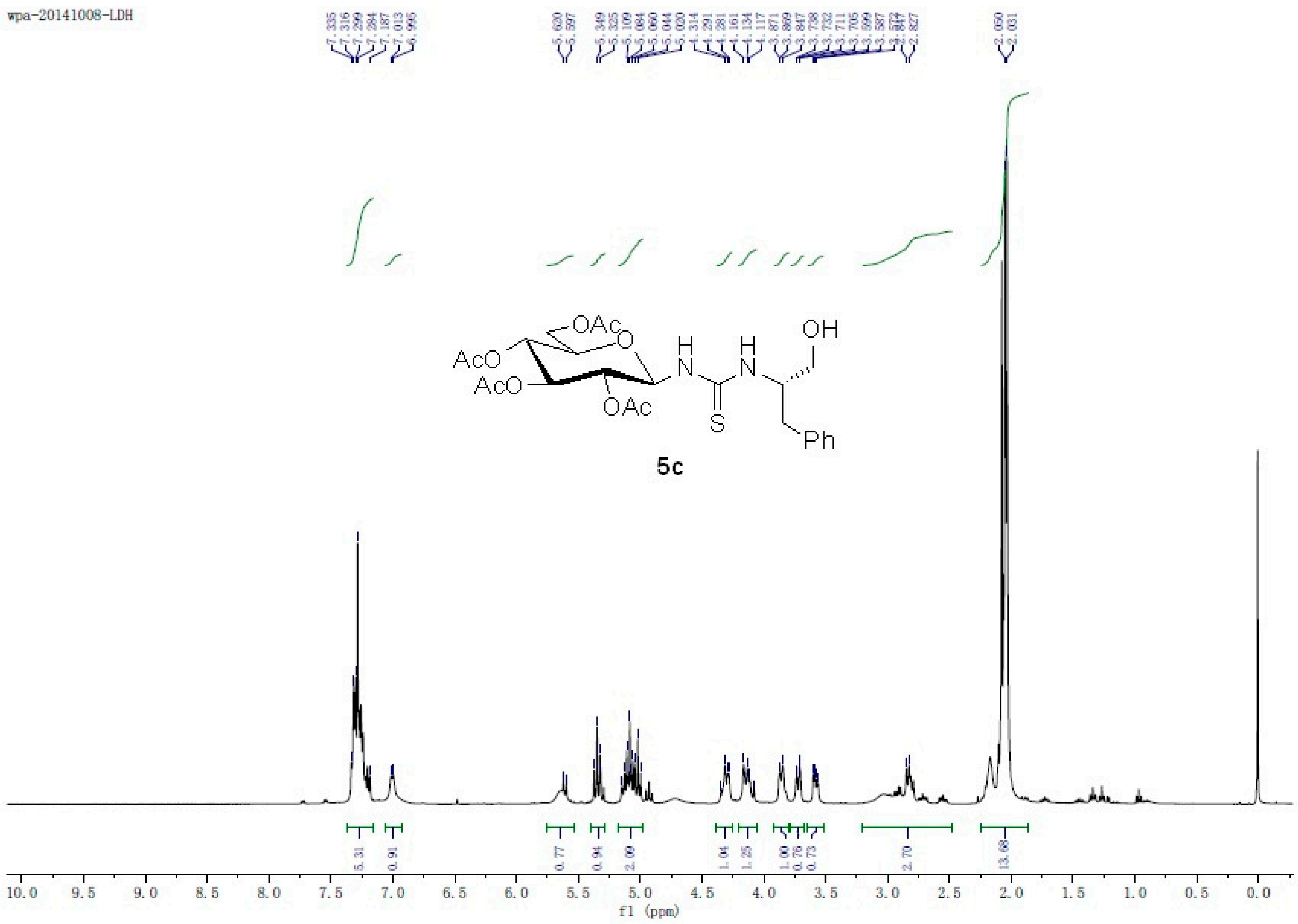
wpa-20141008-LDH
AcO
AcO
OAc
OAc
O
H
N
H
N
S
OH
Ph
5c
f1 (ppm)

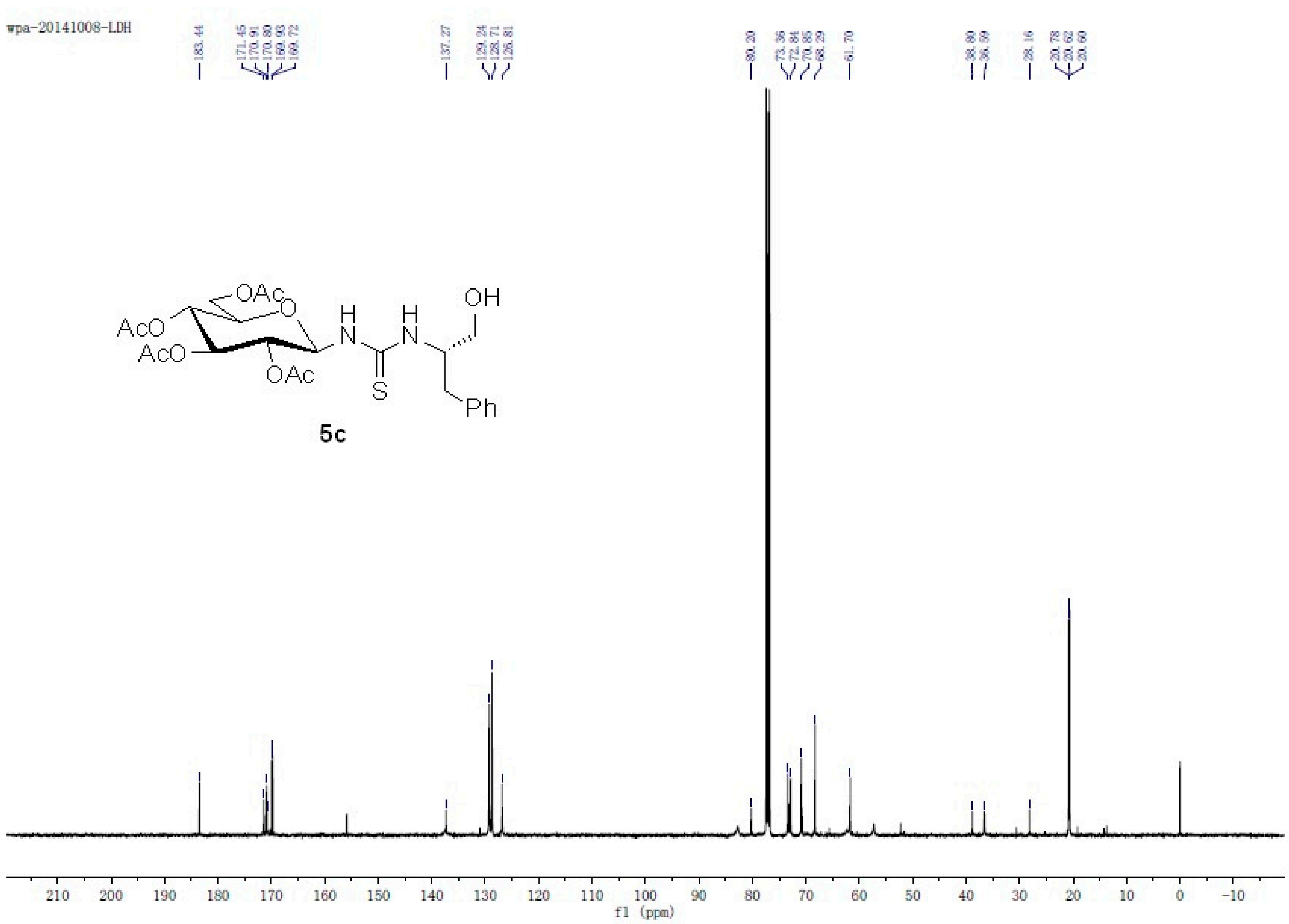
wpa-20141008-LDH
183.44
171.45
170.91
170.80
169.93
169.72
137.27
129.24
128.71
126.81
80.20
73.36
72.84
70.85
68.29
61.70
38.80
36.59
28.16
20.78
20.62
20.60
AcO
AcO
OAc
OAc
OH
Ph
S
f1 (ppm)

5c

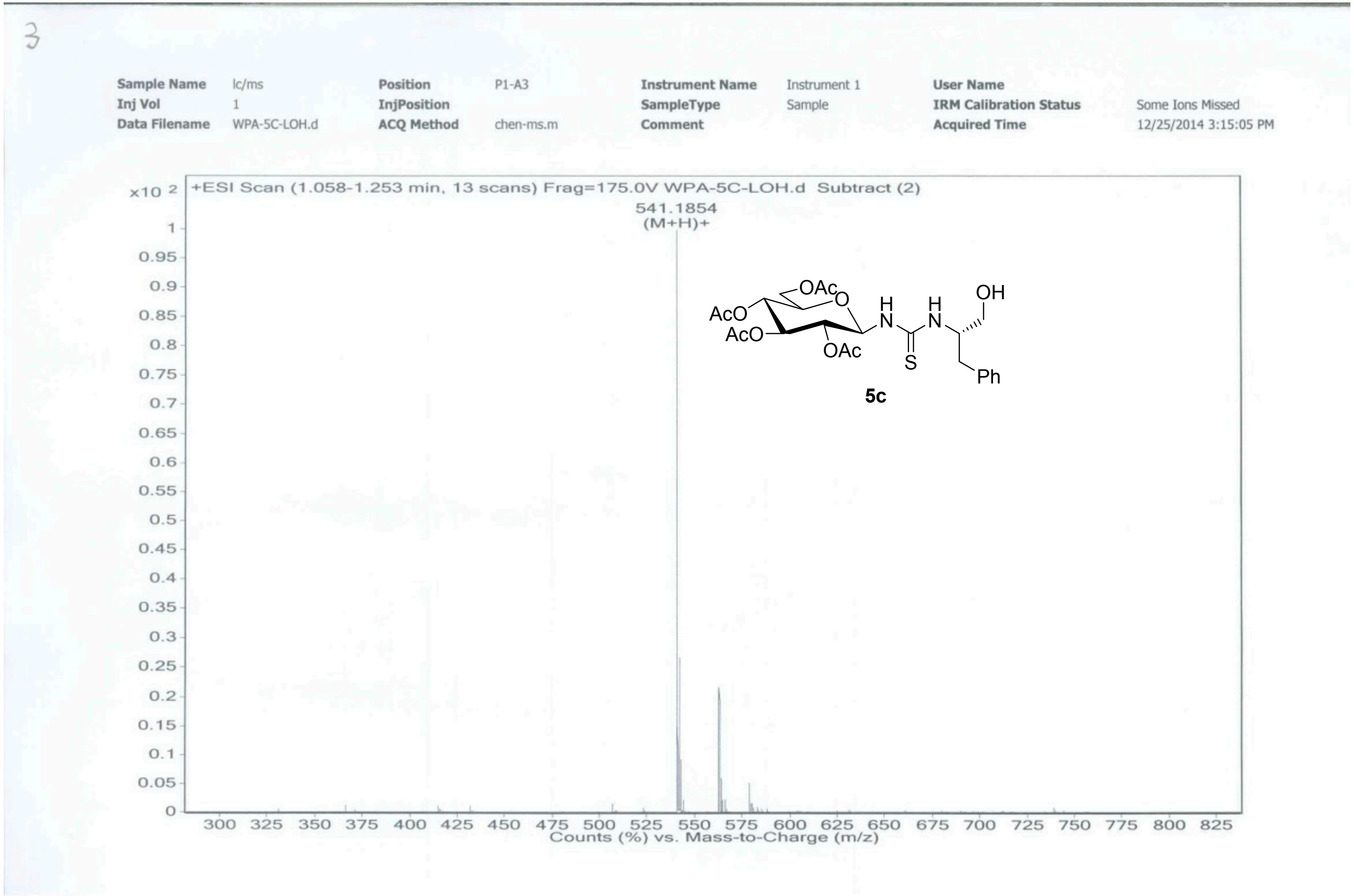

Figure S3. ^{1}H-NMR, ^{13}C-NMR and HRMS of **5c**.

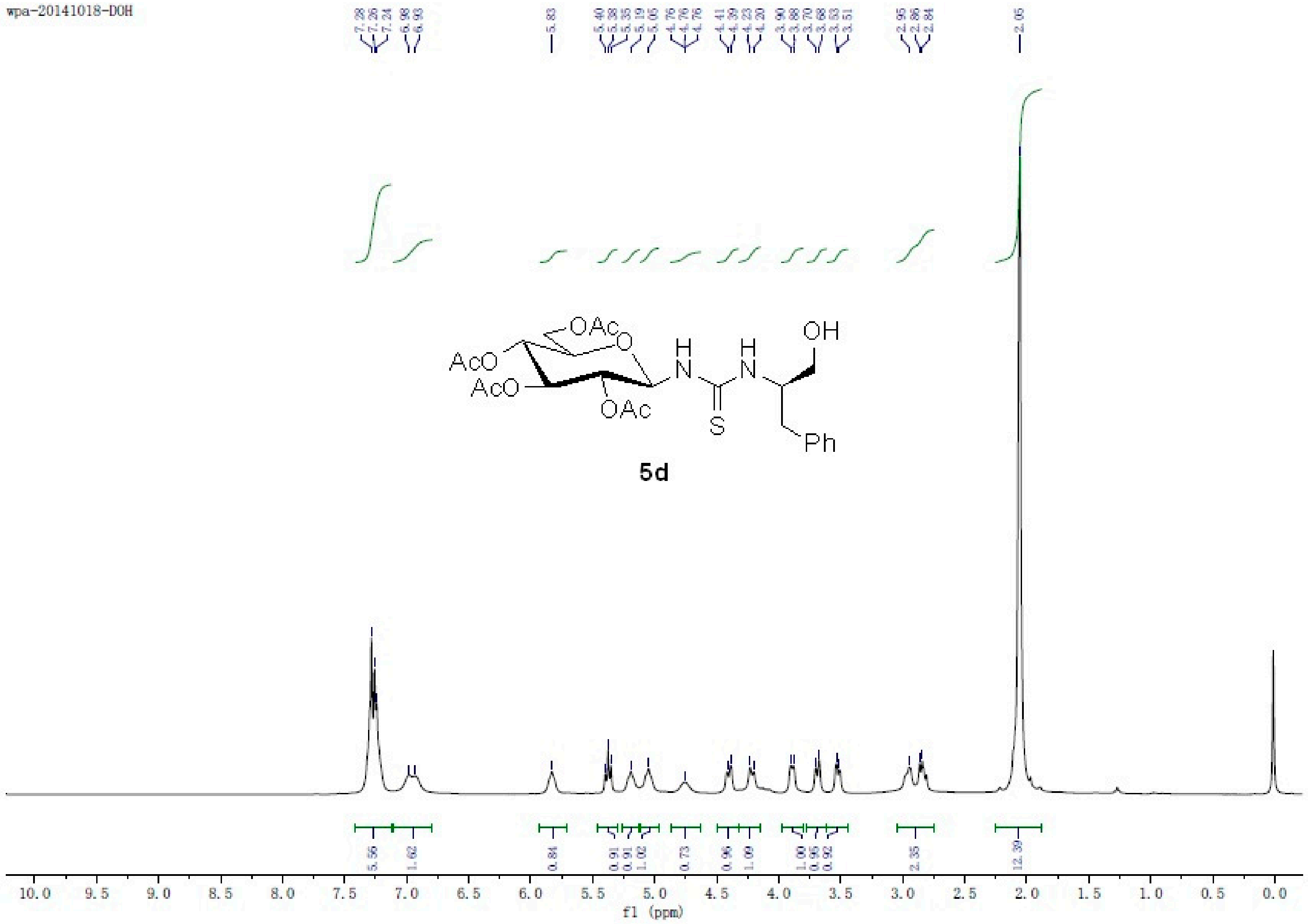
wpa-20141018-DOH
OAc
AcO
AcO
OAc
O
H
N
H
N
S
OH
Ph
5d
10.0 9.5 9.0 8.5 8.0 7.5 7.0 6.5 6.0 5.5 5.0 4.5 4.0 3.5 3.0 2.5 2.0 1.5 1.0 0.5 0.0
f1 (ppm)

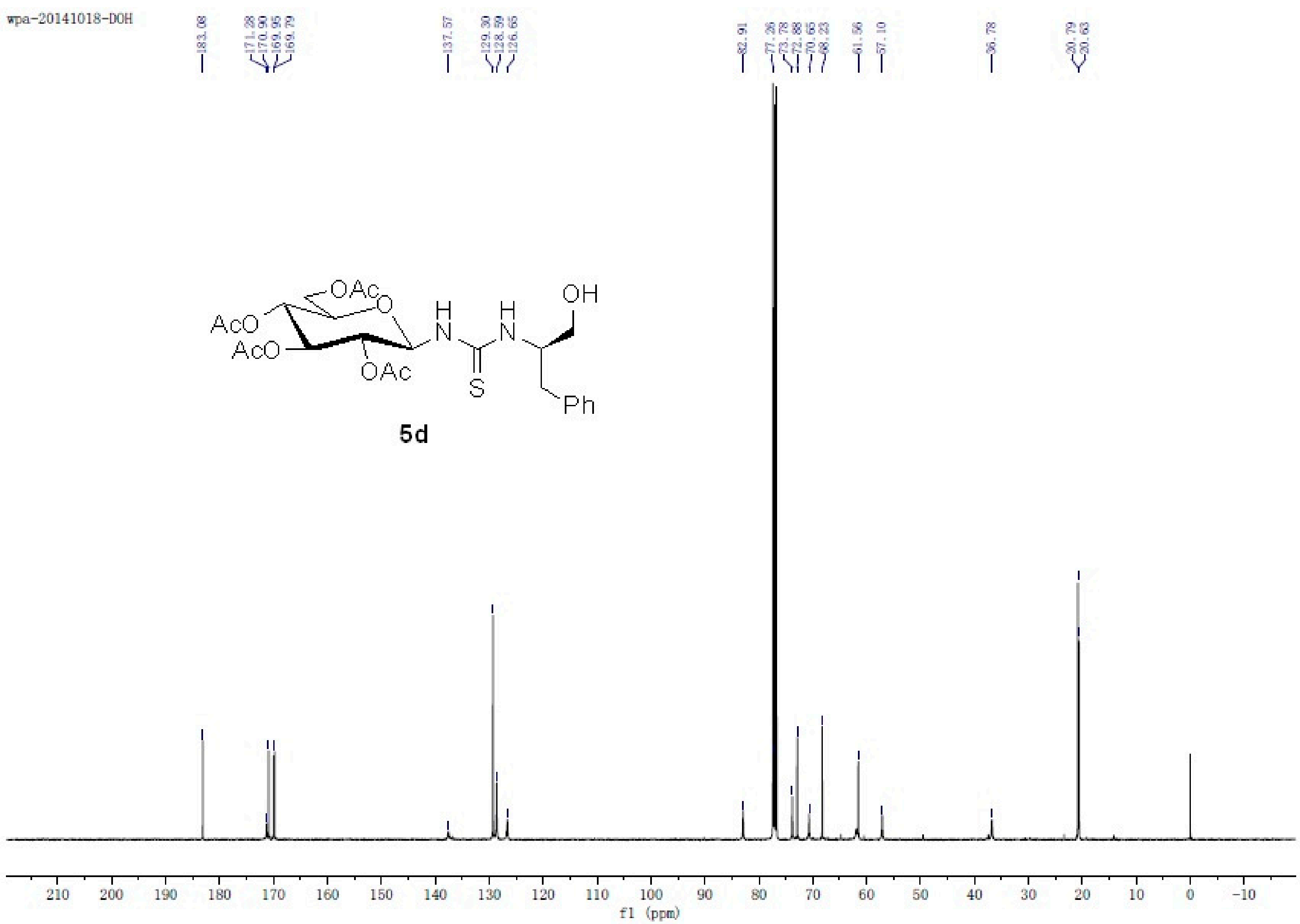
wpa-20141018-DOH
183.08
171.28
170.90
169.95
169.79
137.57
129.30
128.59
126.65
82.91
77.26
73.78
72.88
70.65
68.23
61.56
57.10
36.78
20.79
20.63
AcO
AcO
OAc
OAc
O
H
N
H
N
S
OH
Ph
5d
f1 (ppm)

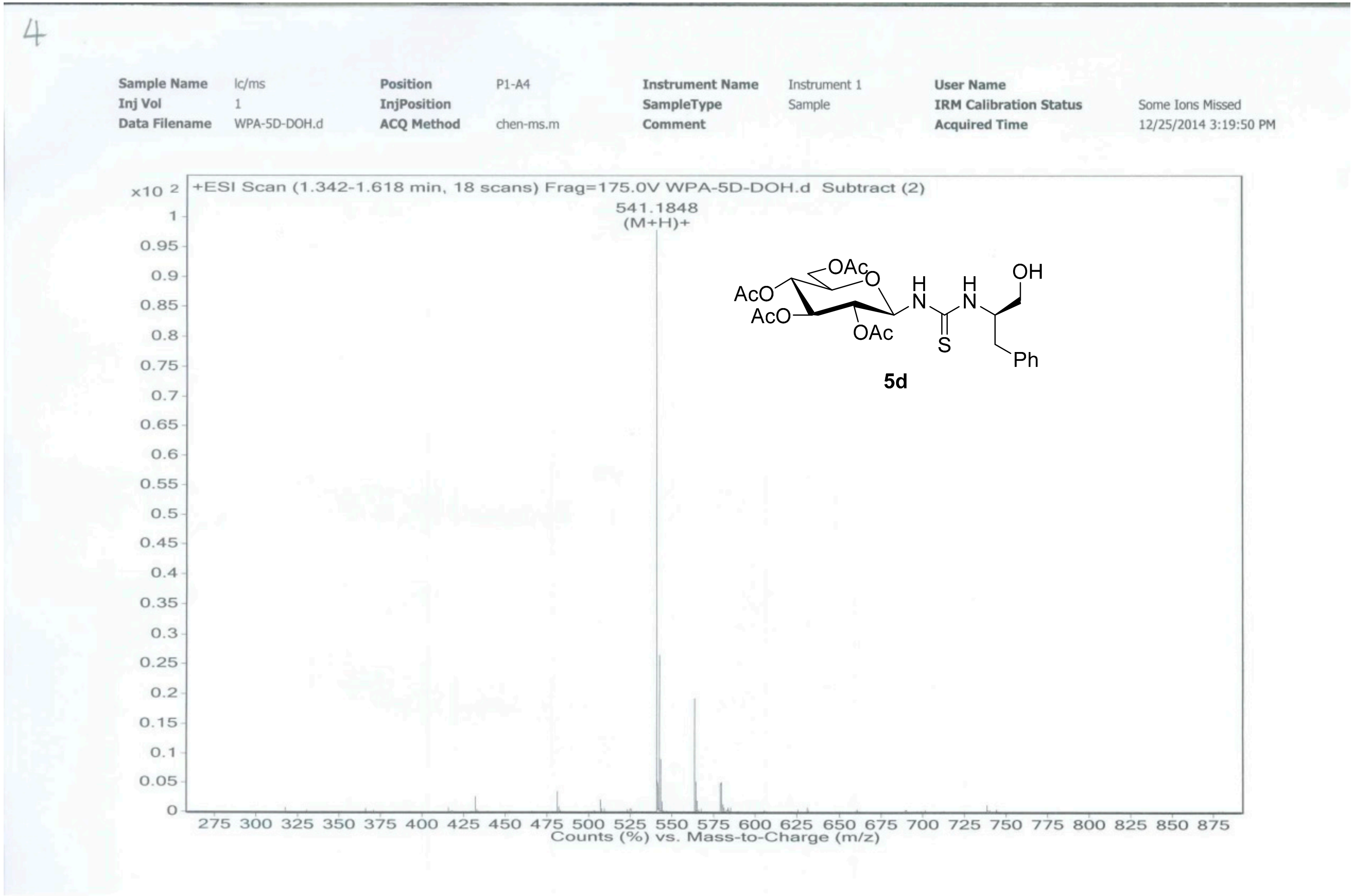

Figure S4. ^{1}H-NMR, ^{13}C-NMR and HRMS of **5d**.

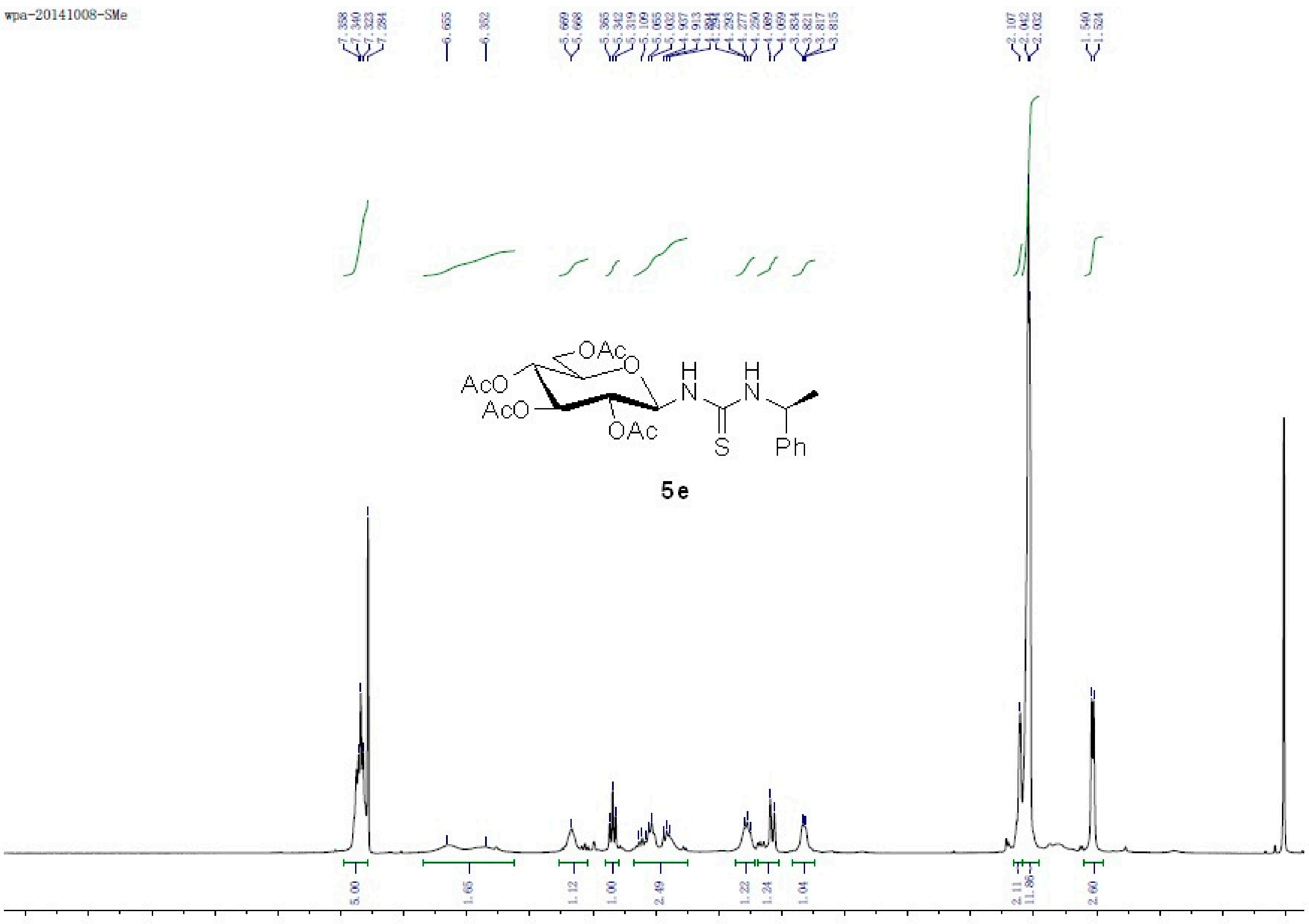
wpa-20141008-SMe
AcO
AcO
OAc
OAc
O
H
N
H
N
S
Ph
5e
5.00
1.65
1.12
1.00
2.49
1.22
1.24
1.04
2.11
11.86
2.60

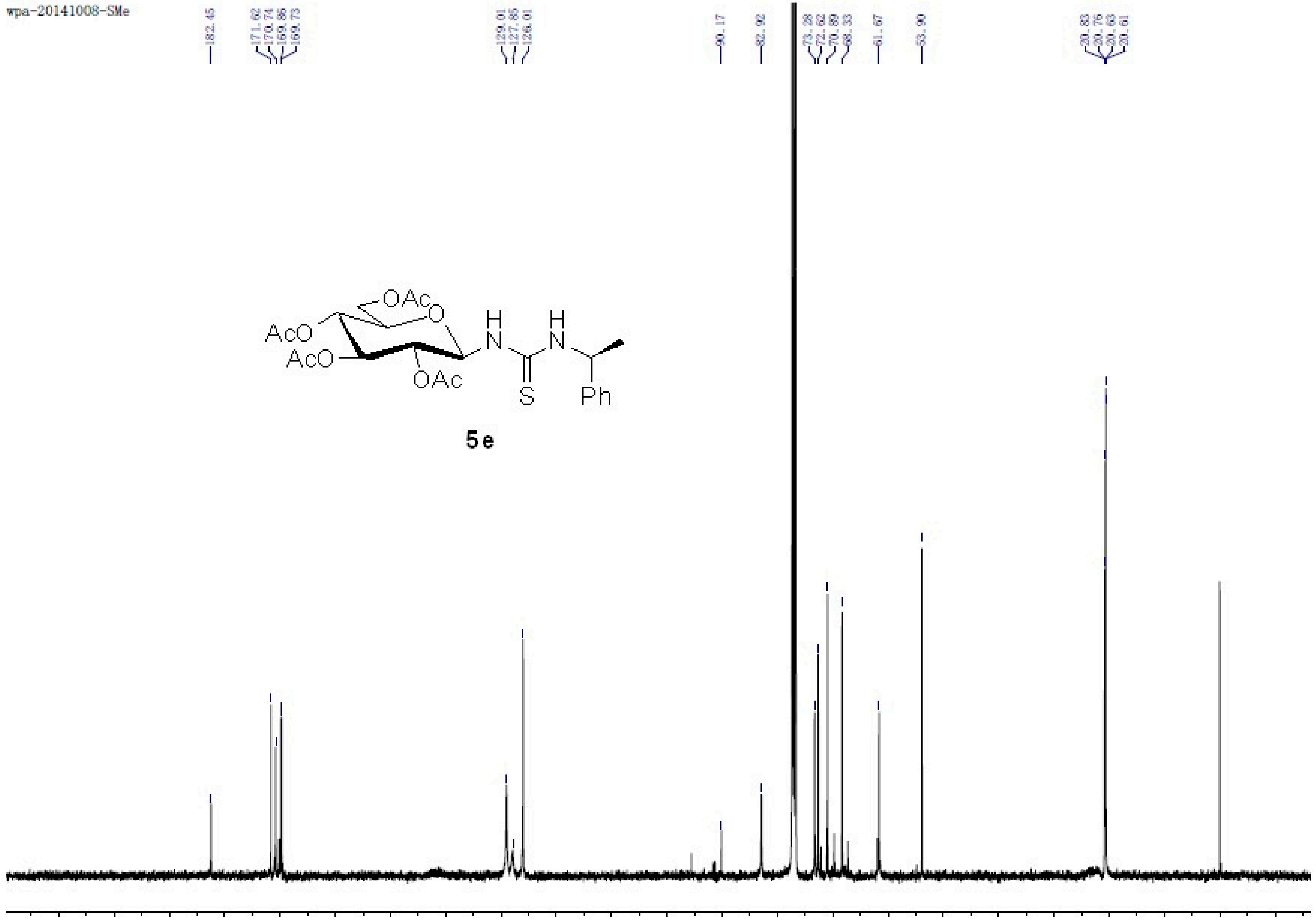
wpa-20141008-SMe
182.45
171.62
170.74
169.85
169.73
129.01
127.85
126.01
90.17
82.92
73.28
72.62
70.89
68.33
61.67
53.90
20.83
20.76
20.63
20.61
OAc
AcO
AcO
OAc
H
N
H
N
S
Ph
5e

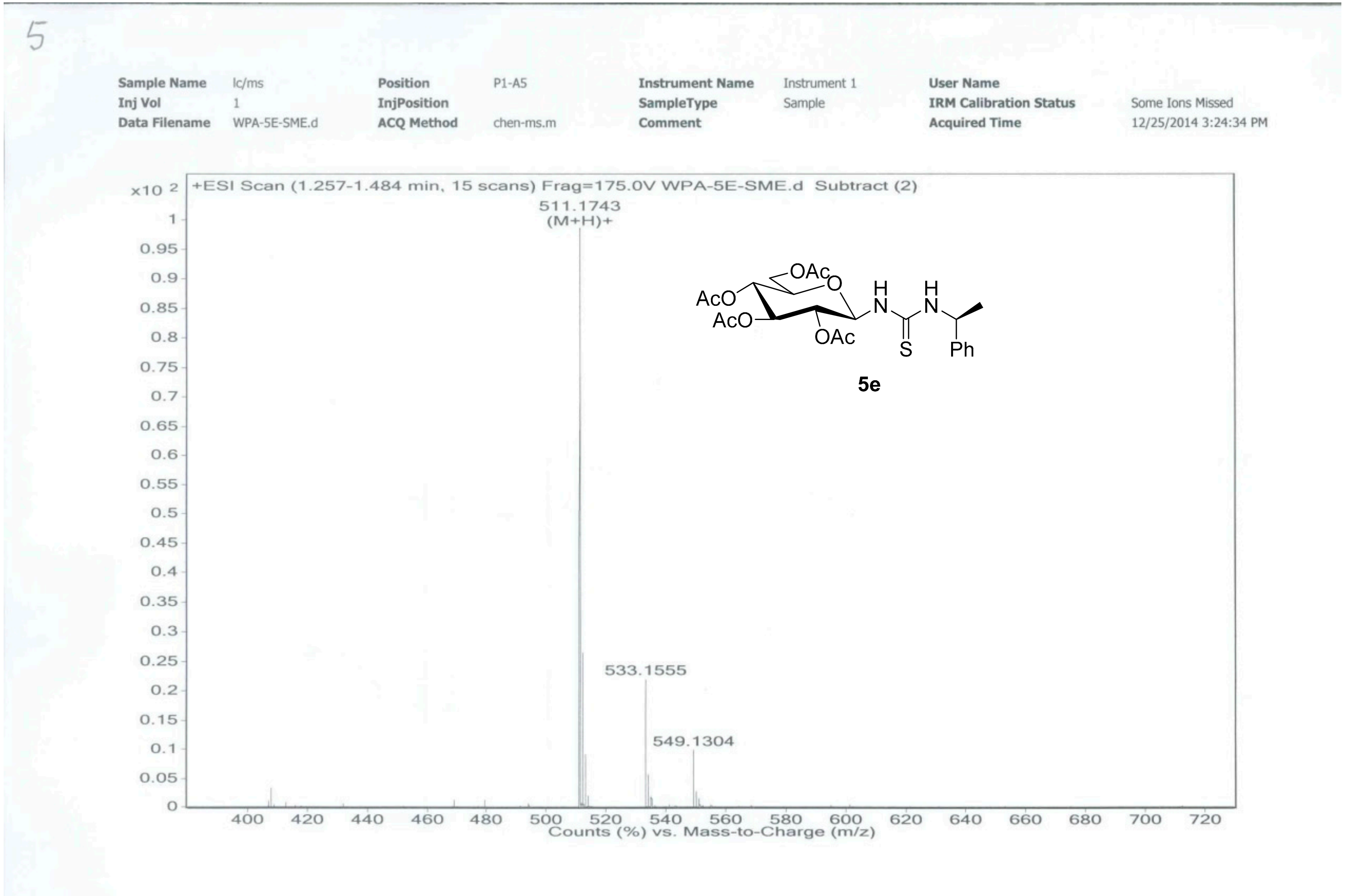

Figure S5. ^{1}H-NMR, ^{13}C-NMR and HRMS of **5e**.

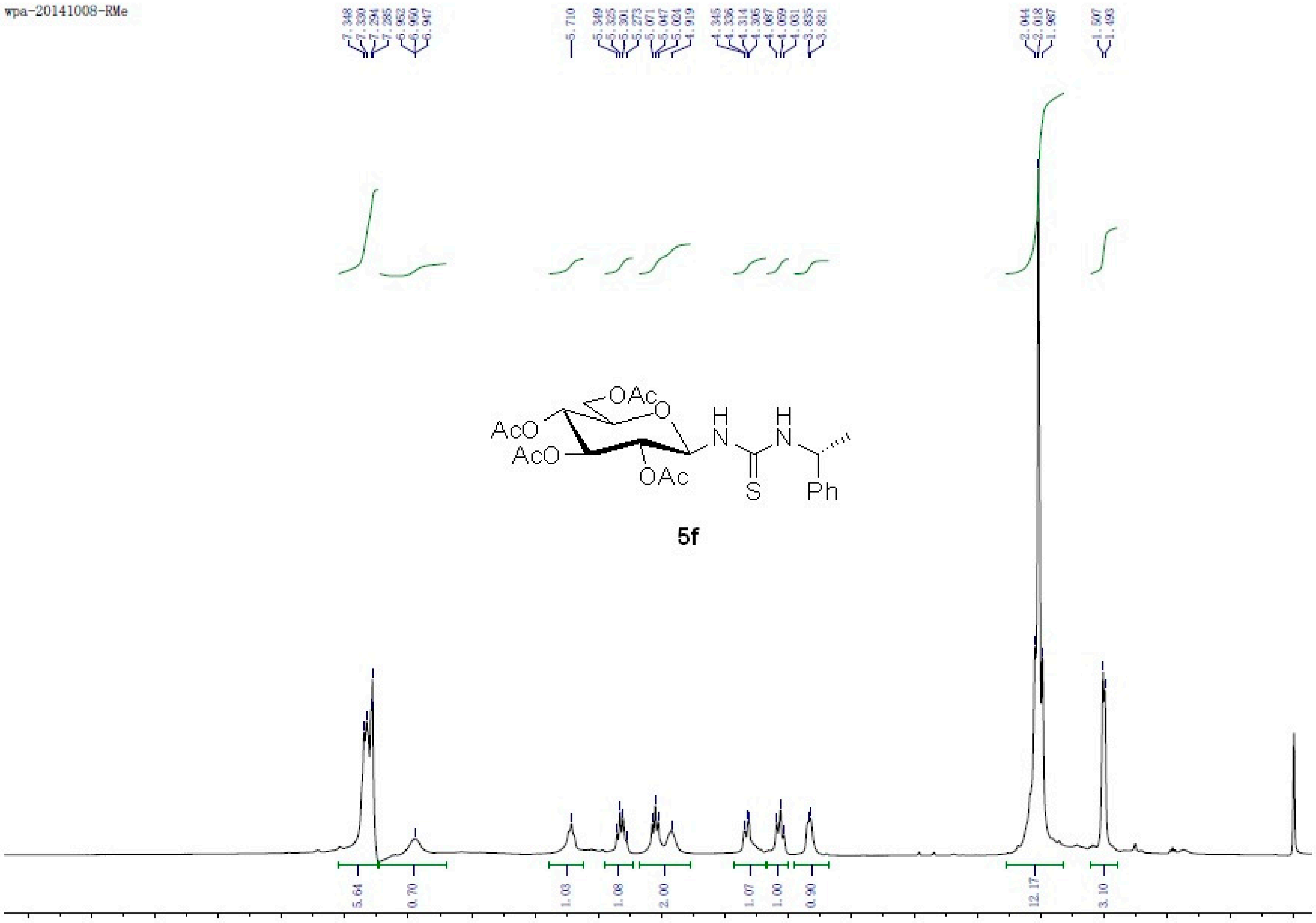
wpa-20141008-RMe
7.348
7.330
7.294
7.285
6.952
6.950
6.947
5.710
5.349
5.325
5.301
5.273
5.071
5.047
5.024
4.919
4.345
4.336
4.314
4.305
4.087
4.059
4.031
3.835
3.821
2.044
2.018
1.987
1.507
1.493
AcO
AcO
OAc
OAc
O
H
N
H
N
S
Ph
5f
5.64
0.70
1.03
1.08
2.00
1.07
1.00
0.90
12.17
3.10

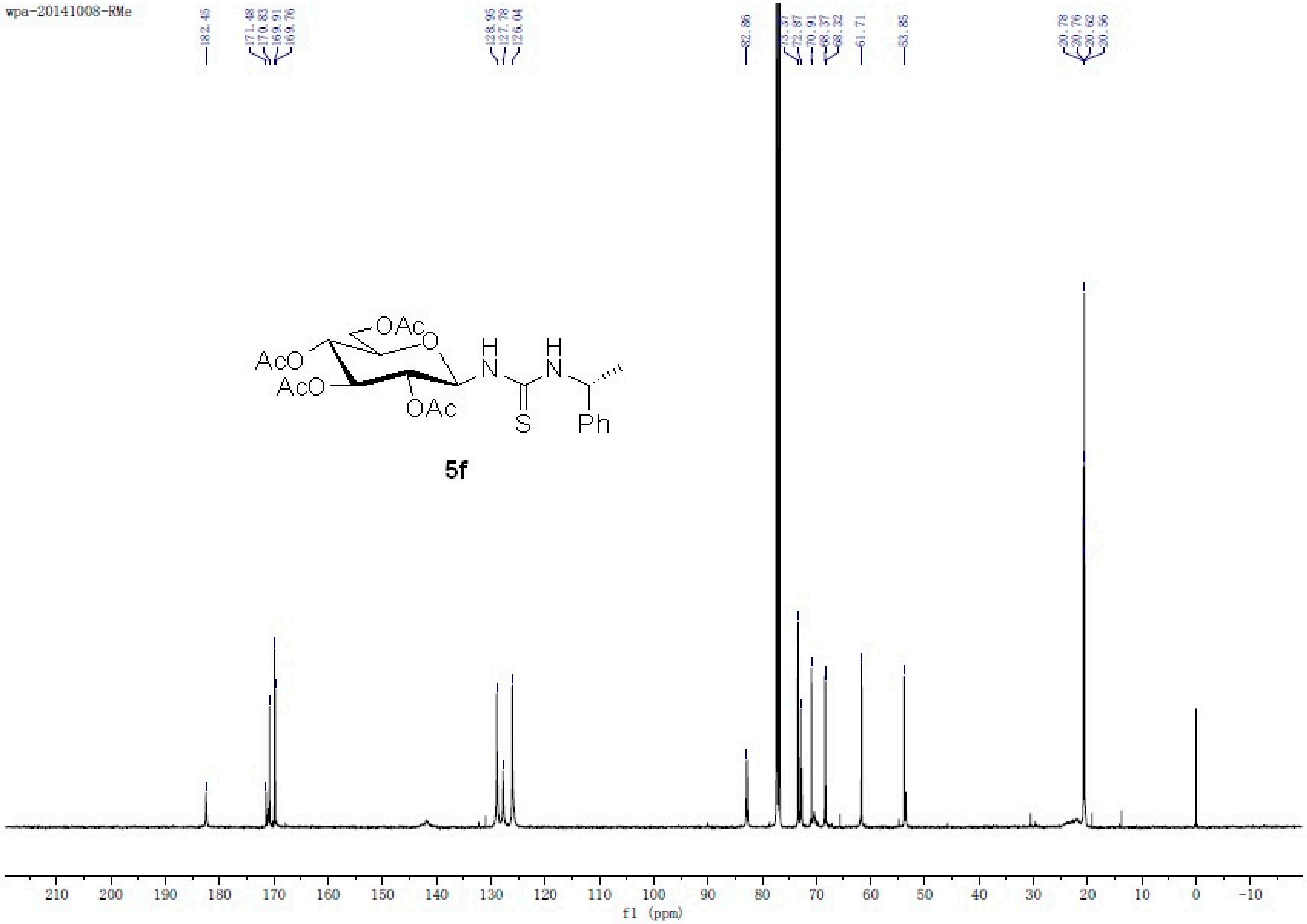
wpa-20141008-RMe
182.45
171.48
170.83
169.91
169.76
128.95
127.78
126.04
82.86
72.87
70.91
68.57
68.32
61.71
53.85
20.78
20.76
20.62
20.56
AcO
AcO
OAc
OAc
O
H
N
H
N
S
Ph
5f
210 200 190 180 170 160 150 140 130 120 110 100 90 80 70 60 50 40 30 20 10 0 -10
f1 (ppm)

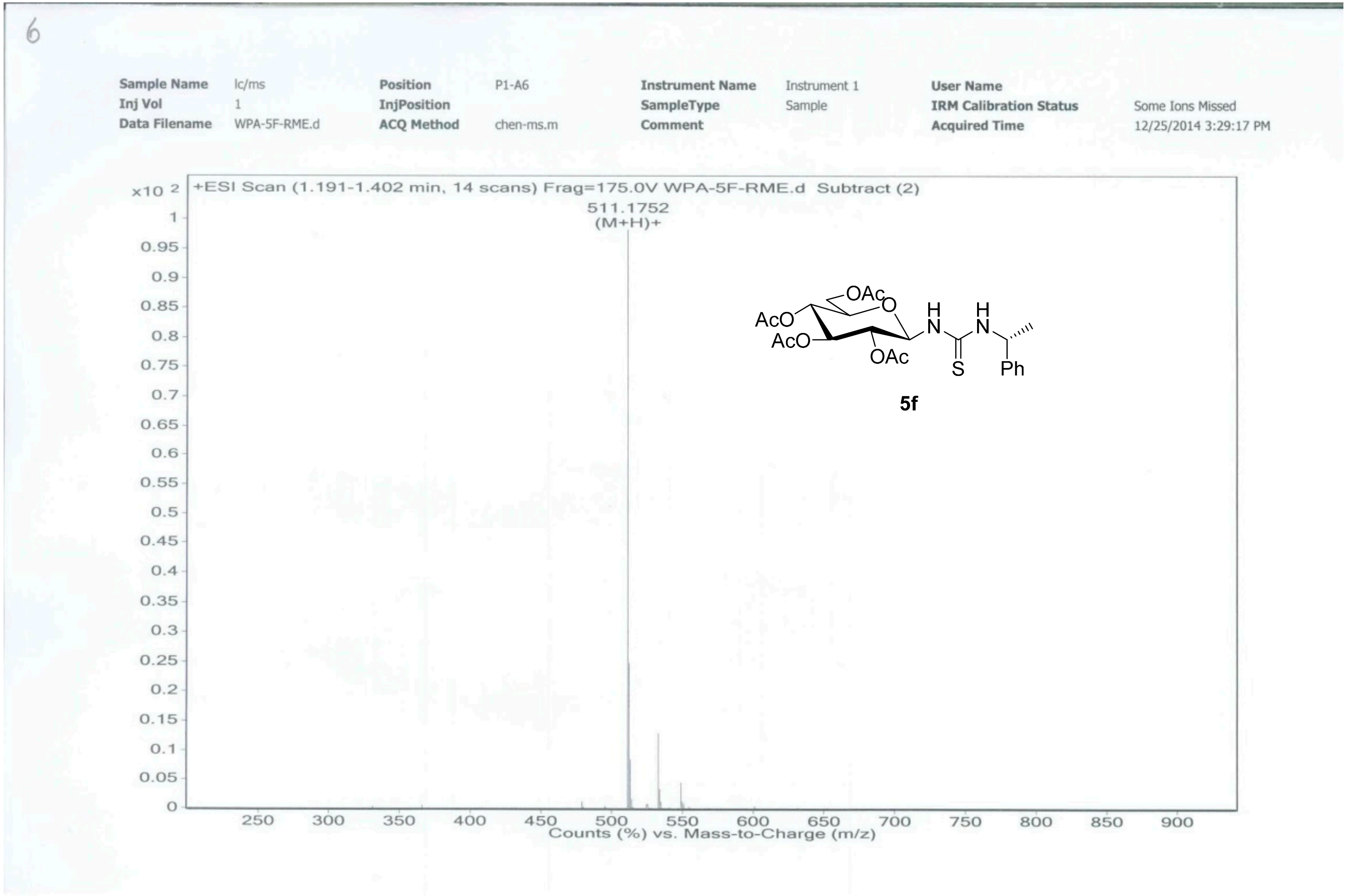

Figure S6. ^{1}H-NMR, ^{13}C-NMR and HRMS of **5f**.

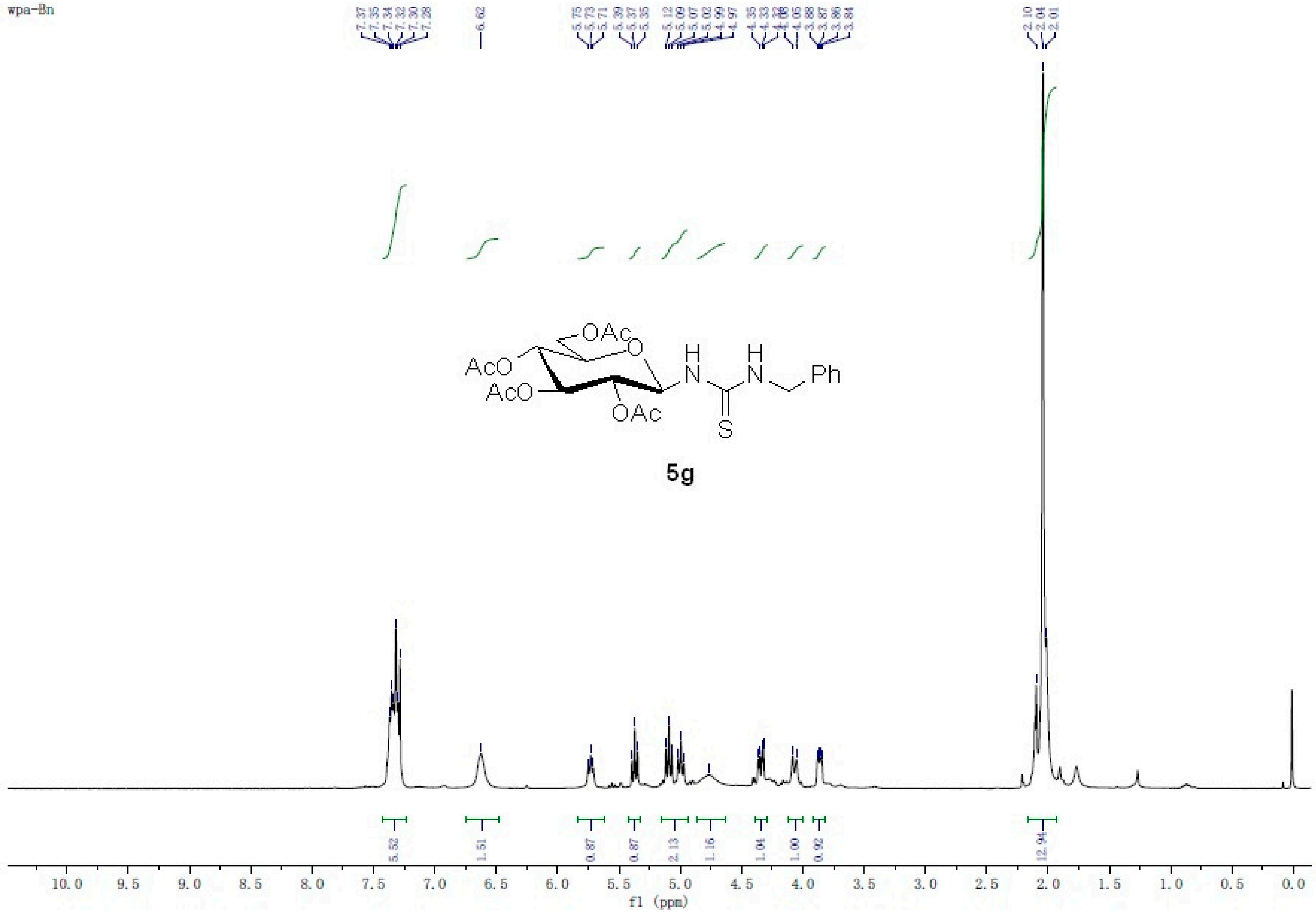
wpa-Bn
AcO
AcO
OAc
OAc
H
N
H
N
Ph
S
5g
f1 (ppm)

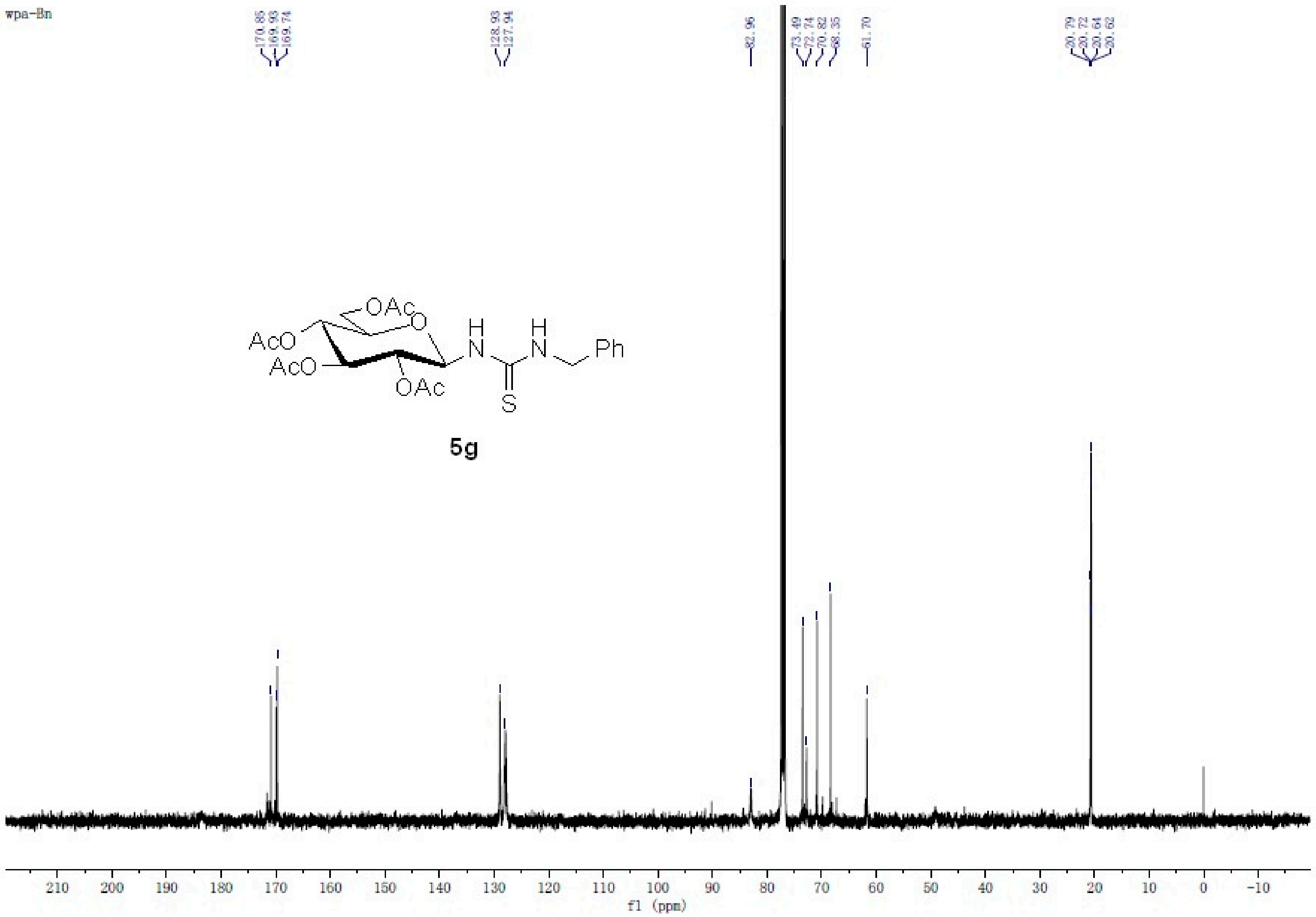
wpa-Bn
170.85
169.93
169.74
128.93
127.94
82.96
73.49
72.74
70.82
68.35
61.70
20.79
20.72
20.64
20.62
AcO
AcO
OAc
OAc
O
H
N
H
N
Ph
S
210
200
190
180
170
160
150
140
130
120
110
100
90
80
70
60
50
40
30
20
10
0
-10
f1 (ppm)

5g

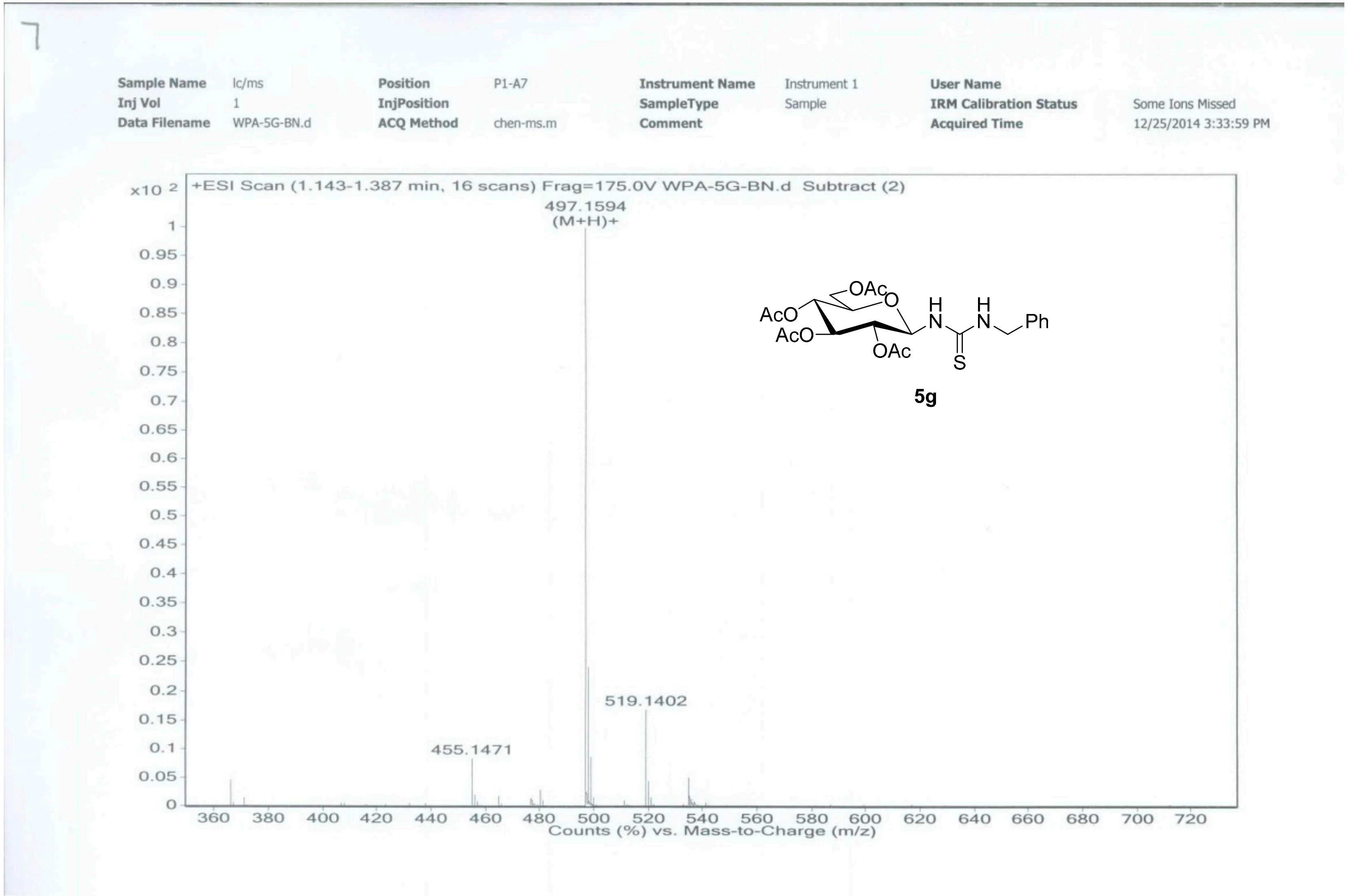

Figure S7. ^{1}H-NMR, ^{13}C-NMR and HRMS of **5g**.

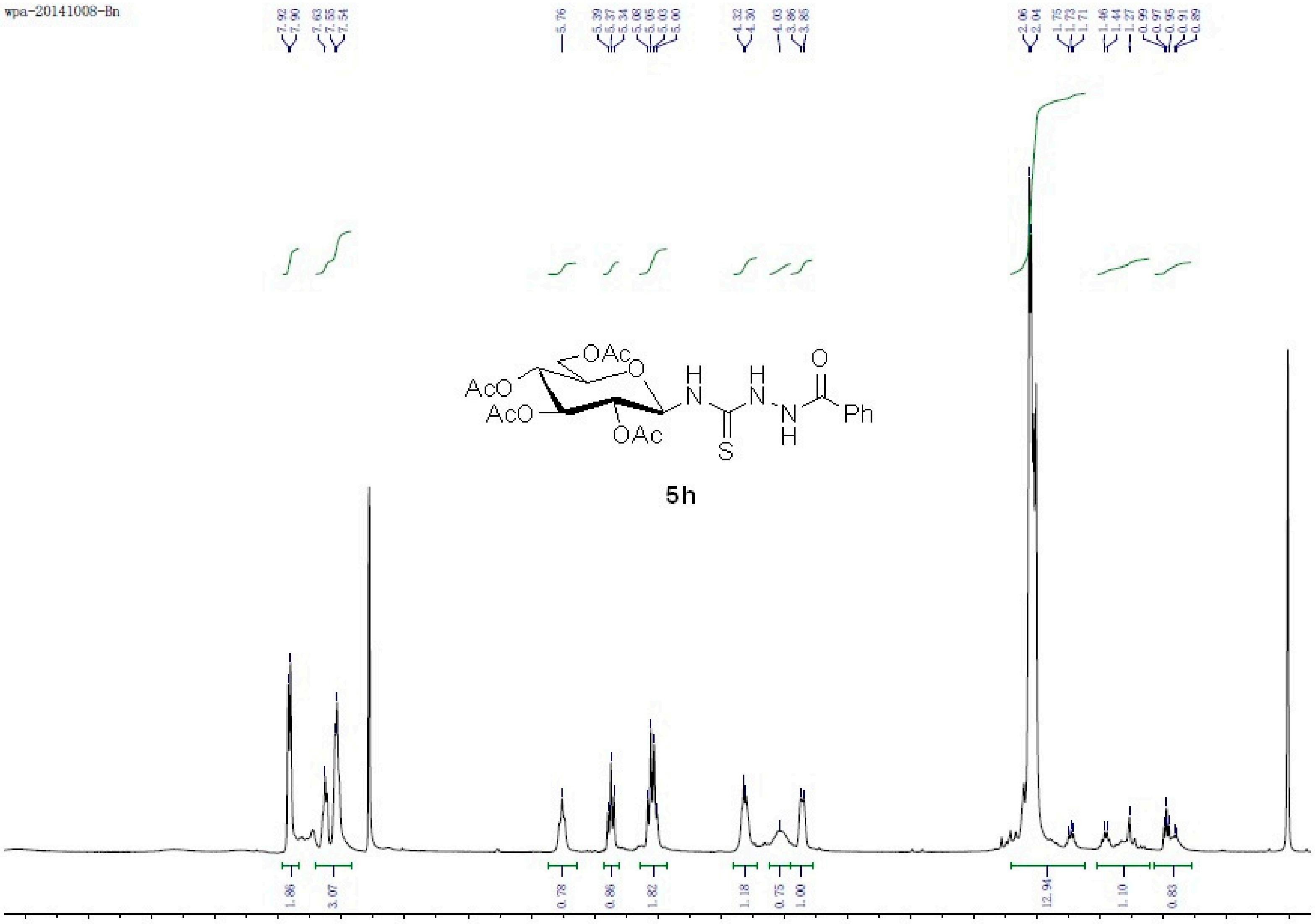
wpa-20141008-Bn
7.92
7.90
7.63
7.55
7.54
5.76
5.39
5.37
5.34
5.08
5.05
5.03
5.00
4.32
4.30
4.03
3.86
3.85
2.06
2.04
1.75
1.73
1.71
1.46
1.44
1.27
0.99
0.97
0.95
0.91
0.89
OAc
AcO
AcO
OAc
O
H
N
H
N
N
H
O
Ph
S
5h
1.86
3.07
0.78
0.86
1.82
1.18
0.75
1.00
12.94
1.10
0.83

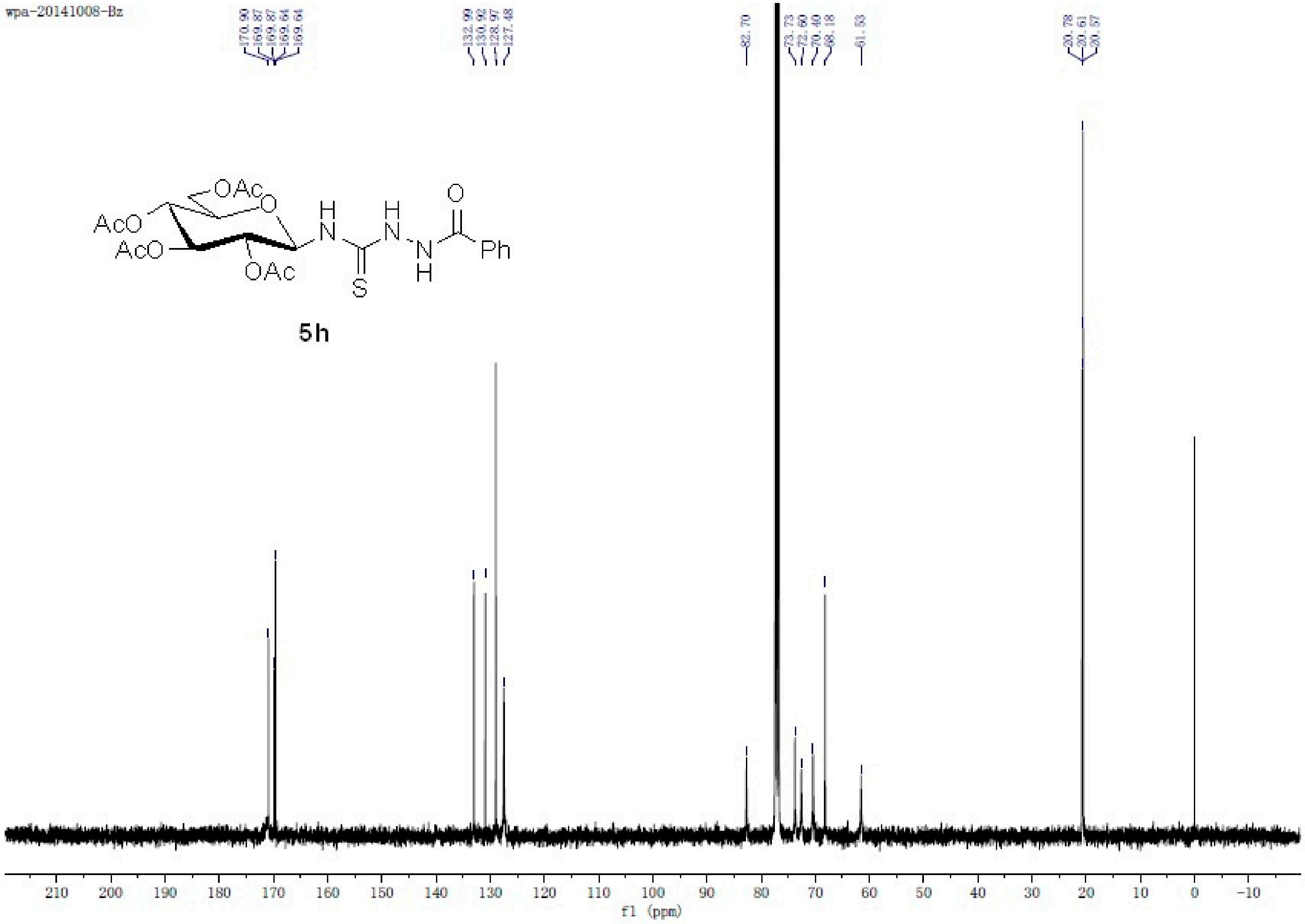
wpa-20141008-Bz
170.90
169.87
169.87
169.64
169.64
132.99
130.92
128.97
127.48
82.70
73.73
72.60
70.49
68.18
61.53
20.78
20.61
20.57
AcO
AcO
OAc
OAc
Ph
5h
f1 (ppm)

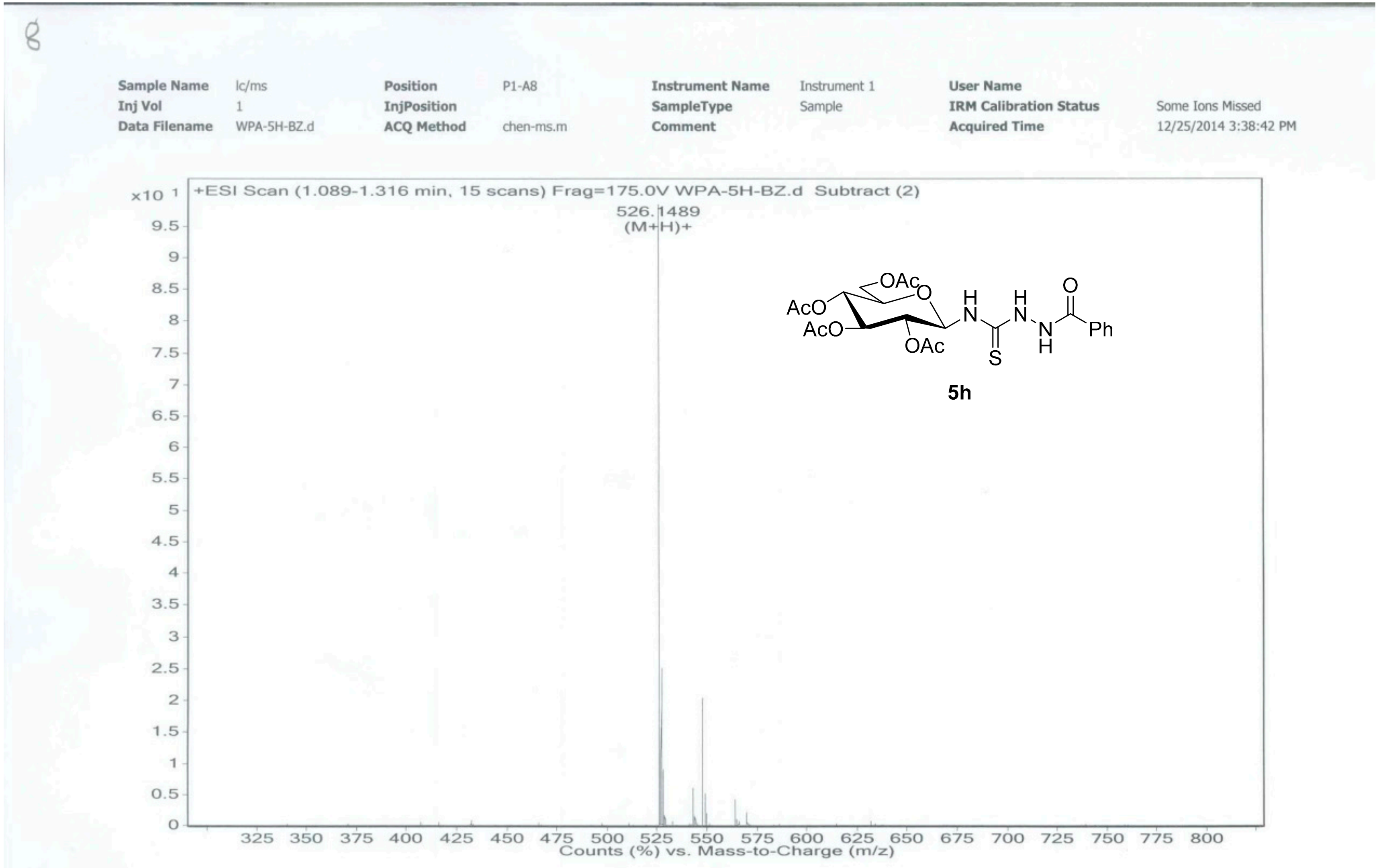

Figure S8. ^{1}H-NMR, ^{13}C-NMR and HRMS of **5h**.

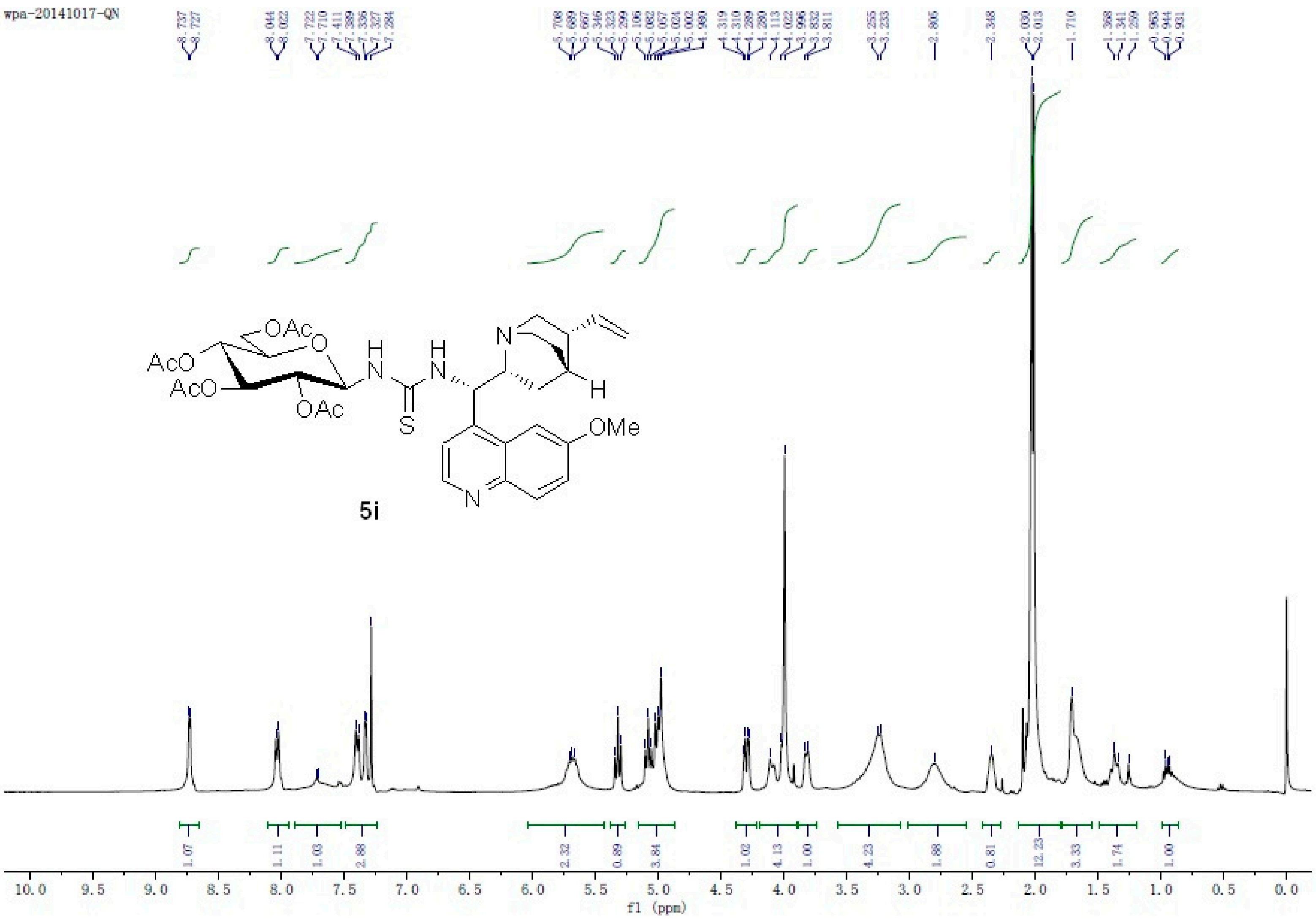
wpa-20141017-QN
AcO
AcO
OAc
OAc
S
OMe
H
5i
f1 (ppm)

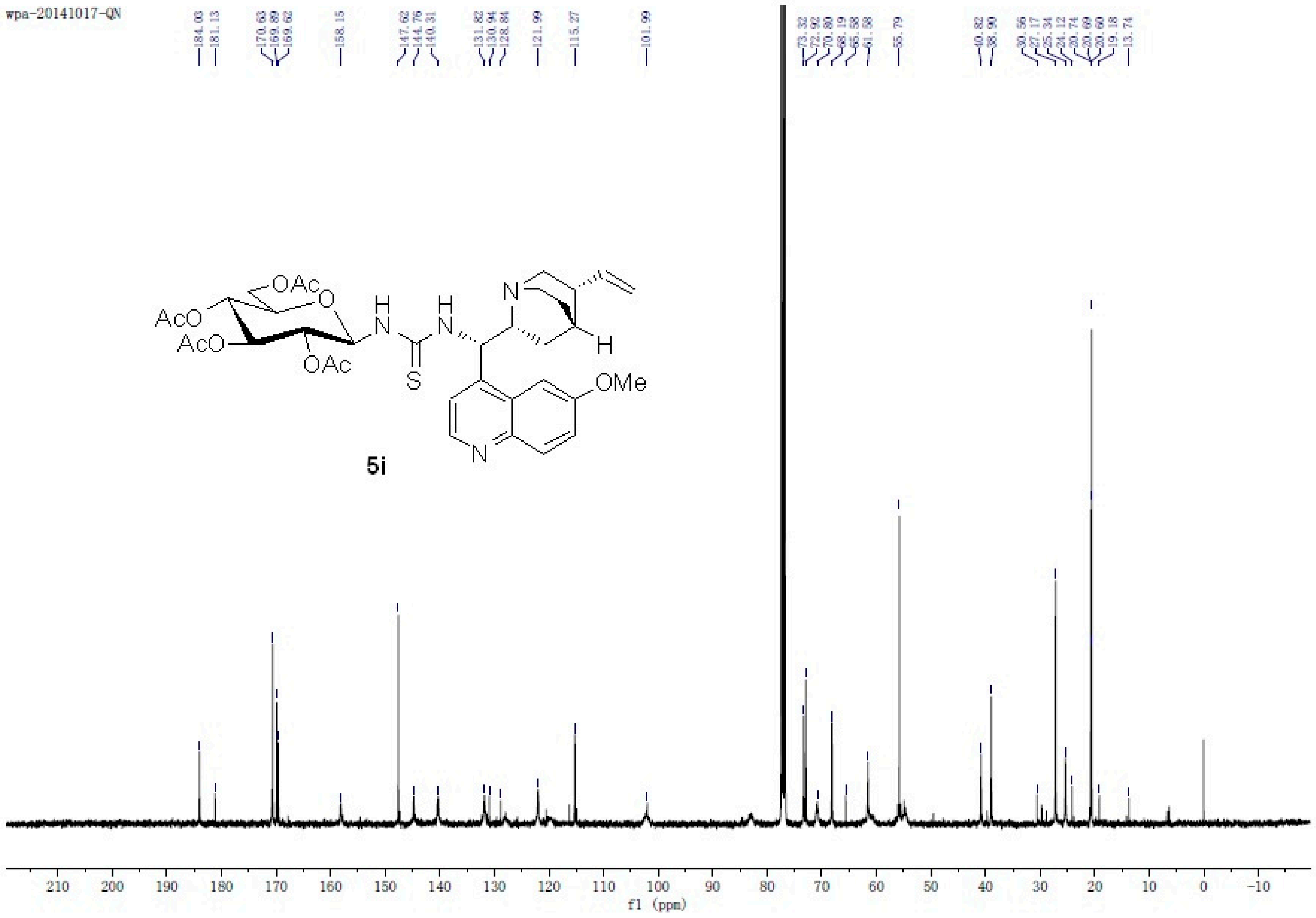
wpa-20141017-QN
184.03
181.13
170.63
169.89
169.62
158.15
147.62
144.76
140.31
131.82
130.94
128.84
121.99
115.27
101.99
73.32
72.92
70.80
68.19
65.88
61.58
55.79
40.82
38.90
30.56
27.17
25.34
24.12
20.74
20.69
20.60
19.18
13.74
AcO
AcO
OAc
OAc
O
H
N
H
N
N
S
H
OMe
N
5i
f1 (ppm)

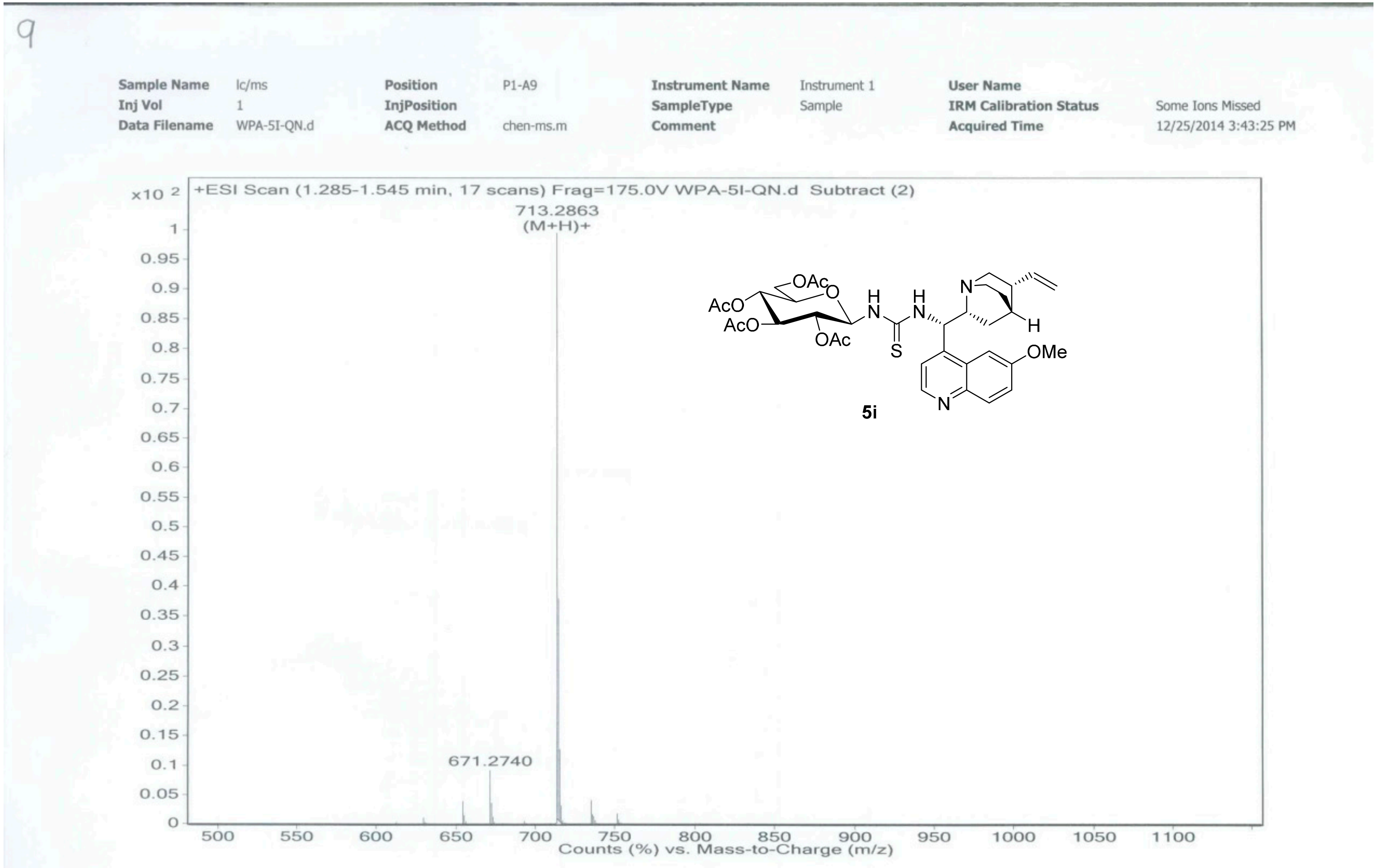

Figure S9. ^{1}H-NMR, ^{13}C-NMR and HRMS of **5i**.

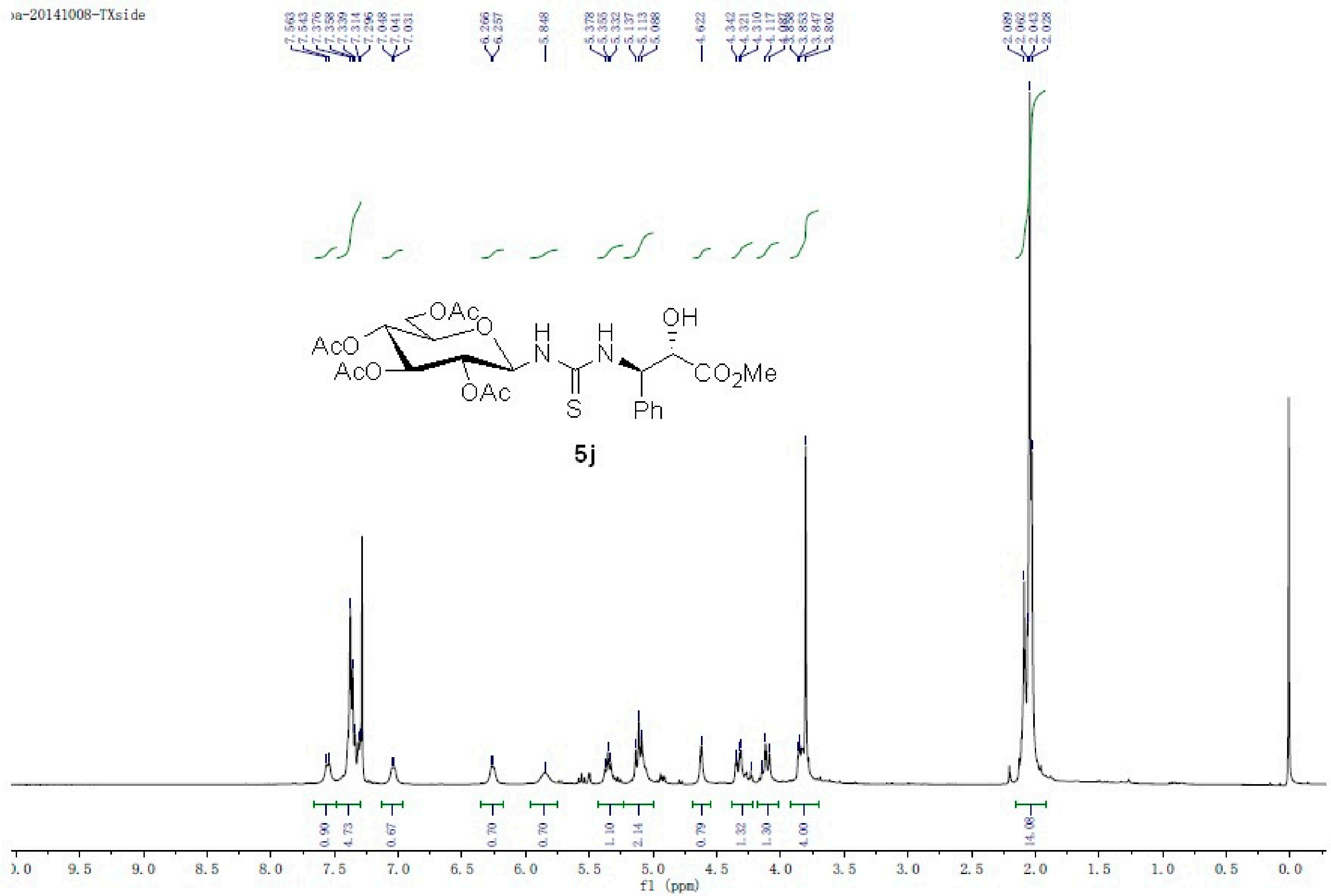
a-20141008-TXside
7.563
7.543
7.376
7.358
7.339
7.314
7.295
7.048
7.041
7.031
6.266
6.257
5.848
5.378
5.355
5.332
5.137
5.113
5.088
4.622
4.342
4.321
4.310
4.117
3.853
3.847
3.802
2.089
2.062
2.043
2.028
AcO
AcO
OAc
OAc
O
H
N
H
N
OH
CO2Me
S
Ph
0.90
4.73
0.67
0.70
0.70
1.10
2.14
0.79
1.32
1.30
4.00
14.08
10.0
9.5
9.0
8.5
8.0
7.5
7.0
6.5
6.0
5.5
5.0
4.5
4.0
3.5
3.0
2.5
2.0
1.5
1.0
0.5
0.0
f1 (ppm)

5j

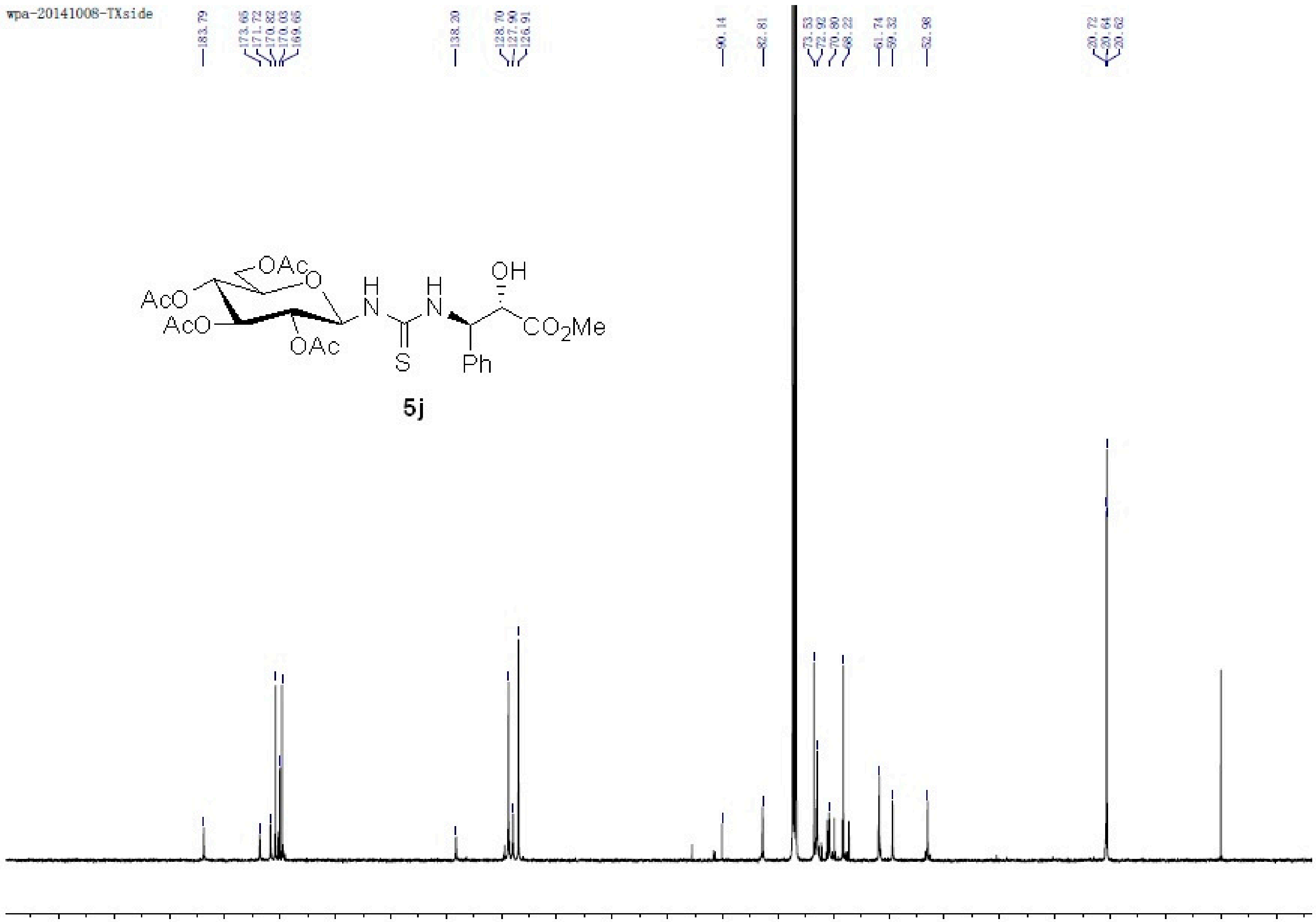
wpa-20141008-TXside
183.79
173.65
171.72
170.82
170.63
169.65
138.20
128.70
127.90
126.91
90.14
82.81
73.53
72.92
70.80
68.22
61.74
59.32
52.98
20.72
20.64
20.62
OAc
AcO
AcO
O
OAc
H
N
H
N
OH
CO2Me
S
Ph
5j

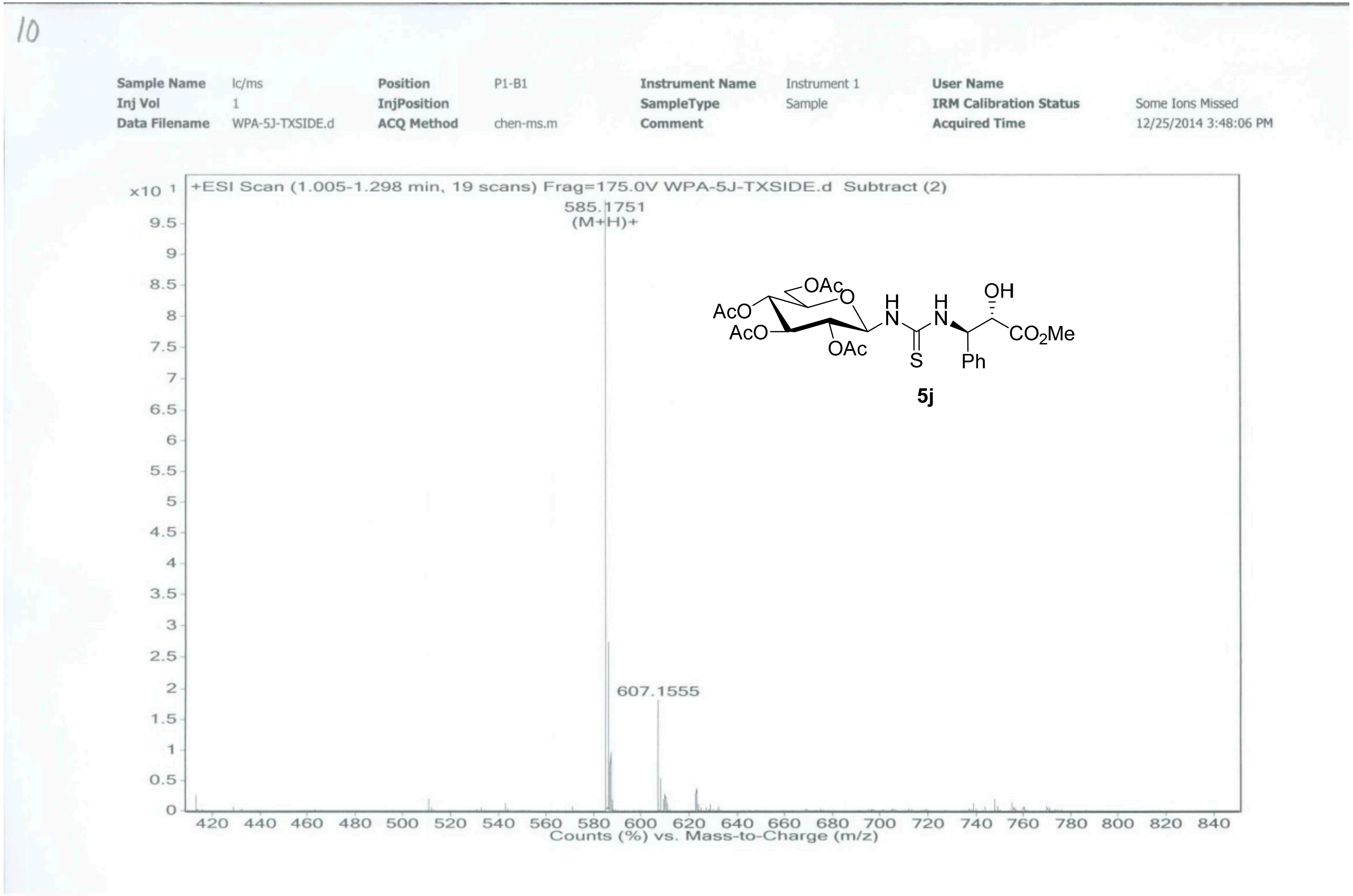

Figure S10. ^{1}H-NMR, ^{13}C-NMR and HRMS of **5j**.
